# A comparison of microbiology and demographics among patients with healthcare-associated, hospital-acquired, and ventilator-associated pneumonia: a retrospective analysis of 1184 patients from a large, international study

**DOI:** 10.1186/1471-2334-13-561

**Published:** 2013-11-27

**Authors:** Andrew A Quartin, Ernesto G Scerpella, Sailaja Puttagunta, Daniel H Kett

**Affiliations:** 1 Division of Pulmonary and Critical Care Medicine, Miller School of Medicine at the University of Miami, Jackson Memorial Hospital, 1611 NW 12th Avenue, C455A, Miami, FL 33156, USA; 2 Department of Veterans Affairs Medical Center, Miami, FL, USA; 3 Jackson Memorial Hospital, Miami, FL, USA; 4 Pfizer Inc, Collegeville, PA, USA

**Keywords:** Nosocomial pneumonia, Healthcare-associated pneumonia, Intensive care, Hospital-acquired pneumonia, Ventilator-associated pneumonia

## Abstract

**Background:**

Acceptance of healthcare-associated pneumonia (HCAP) as an entity and the associated risk of infection by potentially multidrug-resistant (MDR) organisms such as methicillin-resistant *Staphylococcus aureus* (MRSA), *Pseudomonas* and *Acinetobacter* have been debated. We therefore compared patients with HCAP, hospital-acquired pneumonia (HAP), and ventilator-associated pneumonia (VAP) enrolled in a trial comparing linezolid with vancomycin for treatment of pneumonia.

**Methods:**

The analysis included all patients who received study drug. HCAP was defined as pneumonia occurring < 48 hours into hospitalization and acquired in a long-term care, subacute, or intermediate health care facility; following recent hospitalization; or after chronic dialysis.

**Results:**

Data from 1184 patients (HCAP = 199, HAP = 379, VAP = 606) were analyzed. Compared with HAP and VAP patients, those with HCAP were older, had slightly higher severity scores, and were more likely to have comorbidities. *Pseudomonas aeruginosa* was the most common gram-negative organism isolated in all pneumonia classes [HCAP, 22/199 (11.1%); HAP, 28/379 (7.4%); VAP, 57/606 (9.4%); p = 0.311]. *Acinetobacter* spp. were also found with similar frequencies across pneumonia groups. To address potential enrollment bias toward patients with MRSA pneumonia, we grouped patients by presence or absence of MRSA and found little difference in frequencies of *Pseudomonas* and *Acinetobacter*.

**Conclusions:**

In this population of pneumonia patients, the frequencies of MDR gram-negative pathogens were similar among patients with HCAP, HAP, or VAP. Our data support inclusion of HCAP within nosocomial pneumonia guidelines and the recommendation that empiric antibiotic regimens for HCAP should be similar to those for HAP and VAP.

## Background

In 2005, the American Thoracic Society (ATS) and the Infectious Diseases Society of America (IDSA) jointly published guidelines for treatment of nosocomial pneumonia [1]. In addition to patients whose infections met widely used definitions for hospital-acquired pneumonia (HAP) and ventilator-associated pneumonia (VAP), these guidelines identified an additional cohort of patients at risk for potentially multidrug-resistant (MDR) pathogens, those with healthcare-associated pneumonias (HCAP). Criteria for HCAP include pneumonia associated with recent hospitalization in an acute care hospital; residence in a nursing home or extended care facility; or receipt of chronic dialysis, home infusion therapy (including antibiotics), or home wound care. The guidelines suggest that HCAP should be included in the spectrum of HAP and VAP and that patients with HCAP be treated empirically for MDR pathogens [1].

Support for the recommendation that patients with HCAP should receive initial treatment active against MDR pathogens has come predominantly from United States–based studies that documented a high incidence of these pathogens among patients with HCAP [2-8]. Recently, reports from several other countries have also noted increased rates of MDR pathogens in hospitalized patients with HCAP [9-17]. In contrast to these reports, some investigators examining populations of patients hospitalized for HCAP outside of the United States have reported microbiologic patterns more closely resembling those of community acquired pneumonia rather than HAP and VAP [18-21]. This has led some to challenge the use of the HCAP classification itself as well as any associated treatment guidelines [22,23]. Alternatively, the microbiology associated with these infections, and thus the utility of the HCAP category, may vary with geography or healthcare delivery systems.

Given this controversy and the importance of determining the appropriate initial therapy in these seriously ill patients, we analyzed data from a large, international, randomized, double-blind, controlled trial of patients with nosocomial pneumonia and HCAP [24] to compare baseline patient characteristics and microbiology findings (including the relative incidence of infections with potentially MDR pathogens) among patients with HCAP, HAP, or VAP.

## Methods

### Study design

This was a retrospective analysis of data from an international, randomized, double-blind, multicenter trial (ClinicalTrials.gov identifier NCT00084266) that compared the efficacy and safety of linezolid and vancomycin for the treatment of patients with nosocomial pneumonia and HCAP due to methicillin-resistant *Staphylococcus aureus* (MRSA). The details of this trial have been previously reported [24]. Briefly, from October 2004 through January 2010 the study enrolled hospitalized patients aged ≥ 18 years with radiographic and clinical signs of pneumonia consistent with either nosocomial pneumonia or HCAP. The study was approved by an Institutional Review Board or Ethics Committee at each investigational site. The list of investigators and the corresponding Ethics Committees or Institutional Review Boards for this study can be found in an Additional file 1: Figure S1. Written informed consent was obtained from all patients or their legally authorized representative [24]. The intent-to-treat (ITT) population, which included all randomized patients who received ≥ 1 dose of study drug, was used in this analysis. The population analyzed in this study included patients who were later found not to have MRSA infection and who were excluded from the principal analysis in the report of trial results. Of the 156 enrolling centers, 90 were in the United States.

### Pneumonia definitions

Pneumonia was diagnosed by the combination of clinical signs and symptoms, along with a new or evolving infiltrate evident on chest imaging [24]. VAP was defined as onset of pneumonia after > 48 hours of mechanical ventilation, which was calculated by the sponsor from the data available in the case report form. Nosocomial pneumonia cases occurring after at least 48 hours of hospitalization that did not qualify as VAP were classified as HAP. Initially, the study only enrolled patients with pneumonias meeting these criteria. After publication of the ATS/IDSA guidelines in 2005, the study was amended to permit enrollment of patients with HCAP that did not qualify as VAP or HAP. For the trial, a slightly restrictive definition of HCAP was employed: pneumonia acquired in a long-term care or subacute/intermediate healthcare facility (e.g. nursing home, rehabilitation center); pneumonia following recent hospitalization (discharged within 90 days of current admission and previously hospitalized for ≥ 48 hours); or pneumonia in patient who received chronic dialysis care within 30 days prior to study enrollment. This trial did not enroll patients with pneumonia who only met the ATS/IDSA criteria for HCAP by virtue of having recently received home infusion therapy or wound care or of having a family member with an MDR pathogen.

### Assessments

Baseline demographic and clinical data were collected including age, sex, race, and comorbidities. Patients were required to have a baseline respiratory or sputum specimen prior to study enrollment or within 24 hours after first dose of study medication. Microbiologic cultures were performed according to the standard of care at the study site, except for patients with chronic ventilation (> 30 days) or tracheostomy, for whom invasive quantitative cultures were mandated. Patients were followed up to 30 days from the date of study enrollment. In keeping with ATS/IDSA guidelines, we considered MRSA, *Pseudomonas aeruginosa*, and *Acinetobacter* spp. to be potentially MDR pathogens.

### Statistical analysis

All statistical tests were two-sided. To assess statistical differences in the distribution of baseline characteristics between pneumonia groups, one-way analysis of variance was used for continuous variables, and chi-square test was used for categorical variables. P values < 0.05 were considered statistically significant. Statistical procedures were conducted using SAS, version 8.2 (SAS Institute, Inc., Cary, NC, USA).

## Results

The ITT population included 1184 adult patients, of whom 199 presented with HCAP, 379 with HAP, and 606 with VAP. Compared with those with HAP and VAP, patients with HCAP were older and more likely to have diabetes and cardiac, pulmonary, or renal comorbidities (Table 1). HCAP patients also had slightly higher baseline Acute Physiology and Chronic Health Evaluation (APACHE) II scores at the time of diagnosis of pneumonia. Investigators from the United States enrolled 60.2% of all patients in the trial and 87.4% of patients diagnosed with HCAP.

**Table 1:** Baseline characteristics of patients with HCAP, HAP, or VAP

**Baseline characteristic**	**HCAP**	**HAP**	**VAP**	**p value**
	**(n = 199)**	**(n = 379)**	**(n = 606)**	
Age, y, mean (SD)	69.5 (13.4)	63.3 (15.8)	55.8 (19.8)	< 0.001
Male, n (%)	117 (58.8)	247 (65.2)	411 (67.8)	0.067
APACHE II, mean (SD)	18.7 (6.4)	16.1 (6.3)	17.8 (5.7)	< 0.001
Race, n (%)				< 0.001
White	151 (75.9)	217 (57.3)	429 (70.8)	
Black	25 (12.6)	28 (7.4)	72 (11.9)	
Asian	18 (9.1)	97 (25.6)	56 (9.2)	
Other	5 (2.5)	37 (9.8)	49 (8.1)	
Region, n (%)				< 0.001
United States	174 (87.4)	163 (43.0)	376 (62.1)	
Europe	6 (3.0)	51 (13.5)	84 (13.9)	
Latin America	2 (1.0)	43 (11.4)	78 (12.9)	
Asia	14 (7.0)	93 (24.5)	49 (8.1)	
Other	3 (1.5)	29 (7.7)	19 (3.1)	
Comorbidities, n (%)				
Cardiac	153 (76.9)	198 (52.2)	359 (59.2)	< 0.001
Pulmonary	164 (82.4)	186 (49.1)	387 (63.9)	< 0.001
Renal/Urinary	110 (55.3)	127 (33.5)	194 (32.0)	< 0.001
Diabetes	98 (49.3)	128 (33.8)	198 (32.7)	< 0.001
Vascular	74 (37.2)	109 (28.8)	187 (30.9)	0.111
Neoplastic	23 (11.6)	68 (17.9)	42 (6.9)	< 0.001
Hepatobiliary	17 (8.5)	42 (11.1)	91 (15.0)	0.031

*APACHE*, Acute Physiology and Chronic Health Evaluation; *HAP*, Hospital-acquired pneumonia; *HCAP*, Healthcare-associated pneumonia; *VAP*, Ventilator-associated pneumonia.

The distribution of pathogens by pneumonia group is reported in Table 2. The majority of identified organisms were gram-positive, a finding consistent among HCAP, HAP, and VAP patients. Most of these were MRSA [HCAP, 82/199 (41.2%); HAP, 125/379 (33.0%); VAP, 259/606 (42.7%); p = 0.008 for difference between groups]. Gram-negative organisms were cultured from approximately one-third of patients, with *P. aeruginosa* being the most common gram-negative organism in all three pneumonia classes [HCAP, 22/199 (11.1%); HAP, 28/379 (7.4%); VAP, 57/606 (9.4%); p = 0.311]. The other potentially MDR gram-negative species, *Acinetobacter*, was somewhat less common but presented with similar frequencies across pneumonia groups [HCAP, 8/199 (4.0%); HAP, 16/379 (4.2%); VAP, 44/606 (7.3%); p = 0.071]. Most patients had more than one potential pneumonia pathogen cultured, a finding that did not vary with pneumonia type. Among the 689 patients with more than one potential pneumonia pathogen identified, 57.2% had more than one gram-positive species, 5.1% had more than one gram-negative species, and 37.3% had both gram-positive and gram-negative species on culture. Bacteremia rates were similar among pneumonia groups and comparable to rates reported in other series [25,26].

**Table 2:** **Microbiology grouped by HCAP, HAP, and VAP**^a^

**Microbiology**	**HCAP**	**HAP**	**VAP**
	**(n = 199)**	**(n = 379)**	**(n = 606)**
	**n (%)**	**n (%)**	**n (%)**
Gram-positive pathogens	117 (58.8)	226 (59.6)	441 (72.8)
MRSA	82 (41.2)	125 (33.0)	259 (42.7)
MSSA	12 (6.0)	51 (13.5)	107 (17.7)
*Pneumococcus*	4 (2.0)	10 (2.6)	15 (2.5)
Other *Streptococcus* spp.	7 (3.5)	15 (4.0)	18 (3.0)
Gram-negative pathogens	53 (26.6)	113 (29.8)	222 (36.6)
*Pseudomonas aeruginosa*	22 (11.1)	28 (7.4)	57 (9.4)
*Acinetobacter* spp.	8 (4.0)	16 (4.2)	44 (7.3)
*Haemophilus* spp.	6 (3.0)	5 (1.3)	23 (3.8)
*Moraxella catarrhalis*	4 (2.0)	1 (0.3)	2 (0.3)
*Klebsiella* spp.	5 (2.5)	32 (8.4)	41 (6.8)
*Escherichia coli*	10 (5.0)	19 (5.0)	17 (2.8)
*Enterobacter* spp.	3 (1.5)	15 (4.0)	31 (5.1)
*Proteus mirabilis*	1 (0.5)	8 (2.1)	13 (2.1)
*Stenotrophomonas maltophilia*	0 (0)	2 (0.5)	13 (2.1)
Polymicrobial	111 (55.8)	191 (50.4)	387 (63.9)
Culture negative	50 (25.1)	101 (26.6)	79 (13.0)
Bacteremia	28 (14.1)	49 (12.9)	103 (17.0)

*HAP*, Hospital-acquired pneumonia; *HCAP*, Healthcare-associated pneumonia; *MRSA*, Methicillin-resistant *Staphylococcus aureus; MSSA*, Methicillin-susceptible *S. aureus; VAP*, Ventilator-associated pneumonia.

^a^Most commonly isolated pathogens reported (at least 2% in HCAP, HAP, or VAP).

Because the primary focus of the clinical trial was a comparison of therapies for MRSA pneumonia, recruitment efforts may have been directed toward patients thought to be at increased risk for MRSA infection. As a result, the enrolled population may not be representative of the complete HCAP, HAP, and VAP populations where the study was conducted. To address this potential bias, we divided enrolled patients by pneumonia classification and presence or absence of MRSA, comparing the frequencies of *P. aeruginosa* and *Acinetobacter* among the groups (Table 3). Assuming the true population frequencies of *P. aeruginosa* and *Acinetobacter* lie between those observed in the MRSA-infected and non-infected groups, there is little difference by pneumonia classification.

**Table 3:** **Frequency distribution of *Pseudomonas aeruginosa *** **and *Acinetobacter *** **spp. by pneumonia classification and presence or absence of MRSA**

	**HCAP**		**HAP**		**VAP**	
	**No MRSA**	**MRSA**	**No MRSA**	**MRSA**	**No MRSA**	**MRSA**
	**(n = 117)**	**(n = 82)**	**(n = 254)**	**(n = 125)**	**(n = 347)**	**(n = 259)**
	**n (%)**	**n (%)**	**n (%)**	**n (%)**	**n (%)**	**n (%)**
*Pseudomonas aeruginosa*	14 (12.0)	8 (9.8)	18 (7.1)	10 (8.0)	30 (8.6)	27 (10.4)
*Acinetobacter* spp.	5 (4.3)	3 (3.7)	8 (3.1)	8 (6.4)	20 (5.8)	24 (9.3)

*HAP*, Hospital-acquired pneumonia; *HCAP*, Healthcare-associated pneumonia; *MRSA*, Methicillin-resistant *Staphylococcus aureus; VAP*, Ventilator-associated pneumonia.

The all-cause mortality at day 28 was similar among groups [HCAP, 25/199 (12.6%); HAP, 35/379 (9.2%); VAP, 83/606 (13.7%); p = 0.11].

## Discussion

We found that in a population of patients with nosocomial pneumonia enrolled in a large, international, randomized, double-blind trial of therapies for MRSA, the frequencies of potentially MDR gram-negative pathogens were similar among patients with pneumonia classified as HCAP, HAP, or VAP. This suggests that, as recommended in ATS/IDSA guidelines [1] empiric antibiotic regimens utilized for patients hospitalized with HCAP should be similar to those for HAP and VAP.

It is widely accepted that pneumonia occurring after initiation of mechanical ventilation should initially be treated with antibiotics active against MDR pathogens. The rationale is straightforward: ventilated patients are cared for in settings with high antibiotic utilization and often receive antibiotics for other reasons. Both factors contribute to the selection of MDR pathogens when pneumonia occurs. Epidemiologic data in turn provide empiric support for these recommendations [27,28]. Though these rationales and supporting epidemiologic data are somewhat less compelling for pneumonias acquired in the hospital under circumstances other than mechanical ventilation, the extrapolation of VAP regimens to HAP patients has been widely recommended [1,29,30] and generally accepted.

In contrast, recommendations to use antibiotic combinations originally chosen for VAP for patients with HCAP have met with more controversy [19], with some arguing that the HCAP classification itself lacks utility [22]. Our findings speak to both questions. Patients with HCAP were similar to those with HAP and VAP in several key respects: severity of illness; microbiology, particularly the frequency of potentially MDR pathogens; incidence of bacteremia; and short-term mortality. On the other hand, the higher burden of chronic conditions observed among HCAP patients in this study may justify its being a separate classification, particularly for investigators examining factors other than pathogen distribution.

Our study has several limitations. Most importantly, rather than a survey of incident pneumonias, our data derive from a population recruited because of its perceived MRSA risk. Investigators may have taken into consideration factors not accounted for in the collected data that differentiate enrolled patients from other patients with VAP, HAP, and HCAP; e.g. airway specimen gram stain results, history of MRSA colonization, and even infections and colonization of nearby patients. If study investigators intended to enroll patients with MRSA infection, they indeed succeeded, selecting a population with a prevalence of MRSA exceeding that commonly reported [2,31-33]. We feel data from this study therefore should not be used to compare MRSA risk among pneumonia groups. Rather, our analysis focuses on the prevalence of potentially MDR gram-negative organisms, potential pathogens that the study was not seeking, and the agents under study do not treat. Distributions of potentially MDR gram-negative organisms were similar among patients with VAP, HAP, or HCAP and varied little with the presence or absence of MRSA.

That the study design should enhance recruitment of patients with gram-negative pathogens is certainly not obvious. Patients without MRSA were not permitted to complete the clinical trial, and investigator knowledge of certain specific gram-negative risk factors (gram stain results, colonization history, or local ecology) would likely discourage enrollment of patients with gram-negative infections. On the other hand, to the extent that investigators believed that risk factors for MRSA and MDR gram-negative pathogens are similar, efforts to enhance MRSA pneumonia recruitment might also have increased the prevalence of gram-negative pathogens in our sample. In either case, we have little reason to expect that such biases differed by pneumonia class. Our key finding thus seems robust: the likelihood of MDR gram-negative pathogens being present in HCAP is similar to that in HAP and VAP, pneumonias for which coverage of these organisms is widely accepted.

As is always the case in studies that do not obtain tissue to confirm the presence of pneumonia histopathologically, diagnoses and causative microbiology cannot be established with certainty [34]. It is possible that in many cases potentially pathogenic bacteria were merely colonizers, particularly when multiple potential pathogens were found in the same patient. We know of no reason why this would be more likely in HCAP than in HAP or VAP. To the contrary, we suspect colonization is a more frequent phenomenon among patients with VAP, whose airways are instrumented. In any case, distinguishing true pathogens from colonizers in clinical practice is challenging; a commonly adopted strategy is therefore to treat all isolated organisms reasonably likely to be pathogens. Empiric regimens for HCAP should therefore be as broad in spectrum as those for HAP and VAP.

Geography may play an important role in our findings. HCAP patients were enrolled disproportionately in the United States. Possible interpretations include physicians outside the United States not recognizing patients with HCAP as being at risk for MRSA and so not considering them for enrollment; HCAP being more common in the United States than elsewhere; or investigator access to patients with HCAP varying by country. It seems clear that empiric antibiotics for HCAP in the United States should cover MDR pathogens. Given the possible differences in HCAP incidence across geographic regions, we would be hesitant to assume that the microbiology, and hence recommended treatments, should not also vary with location.

## Conclusions

In summary, we compared important demographic characteristics and associated pathogens among patients with HCAP, HAP, or VAP recruited into a large, international pneumonia study. HCAP patients were older and had more comorbidities, higher APACHE II scores, and comparable short-term mortality compared with patients with HAP or VAP. The prevalence of potentially MDR organisms, particularly gram-negatives, was similar across groups, lending support to the recommendation that initial empiric antibiotic therapy should be similar in all groups and should include agents with activity against these pathogens.

## Abbreviations

APACHE: Acute physiology and chronic health evaluation; ATS: American Thoracic Society; HAP: Hospital-acquired pneumonia; HCAP: Healthcare-associated pneumonia; IDSA: Infectious Diseases Society of America; ITT: Intent to treat; MDR: Multidrug-resistant; MRSA: Methicillin-resistant *Staphylococcus aureus*; MSSA: Methicillin-susceptible *Staphylococcus aureus*; VAP: Ventilator-associated pneumonia.

## Competing interests

This study was sponsored by Pfizer Inc. AAQ has no disclosures to report. EGS and SP, formerly of Pfizer, were employees and shareholders of Pfizer Inc at the time this manuscript was developed. DHK has received research support, served as a consultant to, and was on the speakers bureau of Pfizer Inc, Astellas, and GlaxoSmithKline.

## Authors’ contributions

All authors were responsible for conception and design of the study, analysis and interpretation of data, drafting and critical revision of the manuscript, and final approval of the manuscript. EGS and SP were responsible for obtaining funding, acquisition of data, and obtaining administrative, statistical, and technical support. DHK is guarantor of this paper and takes responsibility for the integrity of the work as a whole.

## Pre-publication history

The pre-publication history for this paper can be accessed here:

http://www.biomedcentral.com/1471-2334/13/561/prepub

## Supplementary Material

Additional file 1: Figure S1 Ethics Committees or Institutional Review Boards by Investigator. Click here for file
