# Supplementary material for: A comparison of microbiology and demographics among patients with healthcare-associated, hospital-acquired, and ventilator-associated pneumonia: a retrospective analysis of 1184 patients from a large, international study
Source: BMC Infect Dis. 2013 Nov 27;13:561. doi: 10.1186/1471-2334-13-561 (PMC4222644; doi:10.1186/1471-2334-13-561)
Supplement: Additional file 1: Figure S1 — Ethics Committees or Institutional Review Boards by Investigator. [file 1471-2334-13-561-S1.pdf]

A4 LIST OF INVESTIGATORS AND CORRESPONDING ETHICS COMMITTEES OR INSTITUTIONAL REVIEW BOARDS

Argentina

Coordinating Investigators:
<None Entered>

Center	Principal Investigator	Co-Investigator(s)	Sub-Investigator(s)	Address(es)	Institutional Review Board or Ethics Committee Address(es)
1056 *	Dra. Maria Cristina De Salvo		Dr. Miriam Ines Burgos<br>Dr. Rita Gisela Delgado Vizcarra<br>Ms Florencia Fernandez<br>Dr Ana Maria Molina<br>Dr. Viviana Monica Rodriguez	Hospital General de Agudos "Dr E Tornu", Division Neumotisiologia<br>1er Piso<br>Combatiente de Malvinas 3002<br>Pabellon 3<br>Buenos Aires, 1427<br>ARGENTINA	Comite de Bioetica<br>Hospital General de Agudos Enrique Tornu<br>Combatientes de Malvinas 3002<br>Capitol Federal, 1427<br>ARGENTINA<br><br>Comite Independiente de Etica para Ensayos en Farmacologia Clinica<br>Fundación de Estudios Farmacologicos y de Medicamentos (FEFyM)<br>Uriburu 774- Piso 1°<br>Buenos Aires, 1027<br>ARGENTINA<br><br>Comité de Docencia e Investigación - Hospital General de Agudos "Dr. Enrique Tornu"<br>Combatiente de Malvinas 3002<br>Capital Federal, 1427<br>ARGENTINA

090177e1816ca80c\Approved\Approved On: 06-Oct-2010 15:16

* Did not randomize subjects

Center	Principal Investigator	Co-Investigator(s)	Sub-Investigator(s)	Address(es)	Institutional Review Board or Ethics Committee Address(es)
1058 *	Dr. German Ambasch		Dr. Hilda R. Cabrera<br>Dr. Monica A. Chiarlo<br>MS Maria Cecilia Giavedoni<br>Dr. Miriam A. Maldonado<br>Dr. Marcos Marino<br>Nora B. Peralta	Sanatorio Mayo<br>Belgrano 54<br>Cordoba, 5000<br>ARGENTINA	Comite Independiente de Etica para Ensayos en Farmacologia Clinica<br>Fundación de Estudios Farmacologicos y de Medicamentos (FEFyM)<br>Uriburu 774- Piso 1°<br>Buenos Aires, 1027<br>ARGENTINA<br><br>Comité de Ética Sanatorio Mayo Privado S.A.<br>Belgrano 54<br>Cordoba, 5000<br>ARGENTINA
1059	Dr. Liliana Clara		Dr Martin Rodrigo Ajzenszloz<br>Dr Laura Barcan<br>Dr. Waldo Belloso<br>Dr. Marisa del Lujan Sanchez<br>Dr. Alejandra Valledor	Hospital Italiano<br>Gascon 450<br>Buenos Aires, BUENOS AIRES 1181<br>ARGENTINA	Comite de Etica de Protocolos de Investigacion<br>Hospital Italiano<br>Gascon 450 (C1181ACH)<br>Buenos Aires,<br>ARGENTINA<br><br>Comite Independiente de Etica para Ensayos en Farmacologia Clinica<br>Fundación de Estudios Farmacologicos y de Medicamentos (FEFyM)<br>Uriburu 774- Piso 1°<br>Buenos Aires, 1027<br>ARGENTINA

* Did not randomize subjects

090177e1816ca80c\Approved\Approved On: 06-Oct-2010 15:16

Belgium

Coordinating Investigators:
<None Entered>

Center	Principal Investigator	Co-Investigator(s)	Sub-Investigator(s)	Address(es)	Institutional Review Board or Ethics Committee Address(es)
1110	Prof. Jean-louis Vincent		Dr. Yves Bouckaert<br>Silvia Sanchez Morcillo	Hopital Erasme/Service des Soins Intensifs<br>Route de Lennik 808<br>Brussels, 1070<br>BELGIUM	Ethics Committee Erasme Hospital<br>Ethics Committee Erasme Hospital<br>808, Route de Lennik<br>Brussels, B-1070<br>BELGIUM
1111	Dr. Koenraad Vandewoude		Dominique Benoit<br>Kirsten Colpaert<br>Dr. Jan W. De Waele<br>Dr. Annick De Wolf<br>Johan Decruyenaere<br>Pieter Depuydt<br>Dr. Jan Heerman<br>Dr. Ingrid Herck<br>Dr. Eric Hoste<br>Dr. Joke Nollet<br>Sandra Oeyen<br>Jan Poelaert<br>Carl Roosens<br>Prof. Dirk Vogelaers	Universitair Ziekenhuis Gent - Afdeling Intensieve Zorgen<br>MICU-Kliniekgebouw 12<br>12de verdieping B, De Pintelaan 185<br>Gent, 9000<br>BELGIUM	Ethics Committee Erasme Hospital<br>Ethics Committee Erasme Hospital<br>808, Route de Lennik<br>Brussels, B-1070<br>BELGIUM
1112	Prof. Pierre Damas		Dr. Jean-luc Canivet<br>Dr. Didier Ledoux<br>Dr. Paul Massion<br>Dr. Sonia Piret<br>Dr. Jean-Charles Preiser	Service des soins intensifs Generaux<br>Centre Hospitalier Universitaire de Liege<br>Domaine Universitaire du Sart-Tilman<br>LIEGE 1, B-4000<br>BELGIUM	Ethics Committee Erasme Hospital<br>Ethics Committee Erasme Hospital<br>808, Route de Lennik<br>Brussels, B-1070<br>BELGIUM

090177e1816ca80c\Approved\Approved On: 06-Oct-2010 15:16

Center	Principal Investigator	Co-Investigator(s)	Sub-Investigator(s)	Address(es)	Institutional Review Board or Ethics Committee Address(es)
1185	Dr. Stefaan J. Vandecasteele		Dr. An S. De Vriese<br>Dr. Marc Nauwynck<br>Dr. Achiel Van Hoof	AZ Sint-Jan AV<br>Dept Internal Medicine, Infectious Diseases and Nephrology<br>Ruddershove 10<br>Brugge, 8000<br>BELGIUM<br><br>AZ Sint-Jan AV<br>Department Intensive Care<br>Ruddershove 10<br>Brugge, 8000<br>BELGIUM<br><br>AZ Sint-Jan AV<br>Department Internal Medicine, Hematology<br>ruddershove 10<br>Brugge, 8000<br>BELGIUM	Université Libre de Bruxelles/Hôpital Erasme<br>Comité d'Ethique<br>Route de Lennik 808<br>Bruxelles, 1070<br>BELGIUM

090177e1816ca80c\Approved\Approved On: 06-Oct-2010 15:16

* Did not randomize subjects

090177e1816ca80c\Approved\Approved On: 06-Oct-2010 15:16

Brazil

Coordinating Investigators:
<None Entered>

Center	Principal Investigator	Co-Investigator(s)	Sub-Investigator(s)	Address(es)	Institutional Review Board or Ethics Committee Address(es)
1062 *	Eduardo A. Servolo Medeiros		Ms. Nancy C. J. Bellei<br>Guilherme Henrique Furtado<br>Graziela Lanzara<br>Fernando Gatti Menezes<br>Luiz H. Ota	Hospital São Paulo da Escola Paulista de Medicina - UNIFESP<br>Rua Napoleão de Barros, 690<br>2º andar - Vila Clementino<br>São Paulo, SP 04024-002<br>BRAZIL	Comite de Etica em Pesquisa da Universidade Federal de Sao Paulo - EPM / HSP<br>Rua Botucatu, 572 - Conj. 14 e 41<br>Vila Clementino<br>São Paulo, SP 04023-062<br>BRAZIL
1063 *	Edson Abdala		Patricia Rodrigues Bonazzi<br>Fernanda Betti Maffei<br>Eduardo A. Servolo Medeiros<br>Glaucia Fernanda Varkulja	Hospital das Clínicas da Faculdade de Medicina da Univ. de São Paulo<br>Av. Dr. Arnaldo, 455 - 3º andar<br>Transplante de Fígado<br>São Paulo, SP 01246-000<br>BRAZIL	Comissão de Ética para Análise de Projetos de Pesquisa CAPPesq<br>Rua Dr. Ovídio Pires de Campos, 225 - Sala 505<br>Prédio da Administração<br>São Paulo, SP 05403-010<br>BRAZIL
1065 *	Dr. Clovis Arns da Cunha		Jaime L. L. Rocha<br>Dr. Monica Maria Gomes da Silva<br>Paula Virgina Michelon Toledo	Hospital Vita<br>Rodovia BR 116, Km. 396 - nº 4021<br>Bairro Alto<br>Curitiba, PR 82590-100<br>BRAZIL	Comite de Etica em Pesquisa em Seres Humanos do HC-UFPR<br>Rua General Carneiro, 181<br>Curitiba, PR 80060-900<br>BRAZIL

* Did not randomize subjects

090177e1816ca80c\Approved\Approved On: 06-Oct-2010 15:16

Center	Principal Investigator	Co-Investigator(s)	Sub-Investigator(s)	Address(es)	Institutional Review Board or Ethics Committee Address(es)
1067	Dr. Luis Fernando Aranha Camargo		Silvana Maria Almeida<br>Erika M K Andrade<br>Mauricio Beller Ferri<br>Liou Hsing Ling<br>Dr. Tatiana Mohovic<br>Dr. Rogério H Passos<br>Dr. Adriano J. Pereira<br>Dr. Janaina S Ricardo<br>Dr. Moacyr Silva Junior<br>Ana Maria Beltrami Sogayar<br>Camila Paiva de Vasconcelos	Sociedade Beneficente Israelita Brasileira Hospital Albert Einstein<br>Av. Albert Einstein, 627<br>São Paulo, SP 05651-901<br>BRAZIL	Comite de Etica em Pesquisa do Hospital Israelita Albert Einstein<br>Av. Albert Einstein, 627<br>Morumbi<br>São Paulo, SP 05651-901<br>BRAZIL
1178	Dr. Fernando Gongora Rubio		Dr. Delzi V. N. Gongora<br>Lauriane G. Leite<br>Luana Sousa Marques<br>Dr. Maria G. de Lucca Oliveira<br>Lilian F. Prado<br>Dr. Livia S. Rodrigues<br>Dr. Wilson J. Q. Santos<br>Alcides Pinto de Souza Junior	Hospital de Base ¿ Fundacao Faculdade Regional de Medicina de Sao Jose do Rio Preto (FUNFARME)<br>Av. Brigadeiro Faria Lima, 5544<br>Jd. Universitario<br>Sao Jose do Rio Preto, SP 15090-000<br>BRAZIL	Comite de Etica em Pesquisa da Faculdade de Medicina de Sao Jose do Rio Preto FAMERP<br>Av. Brigadeiro Faria Lima, 5416<br>Jardim Universitario<br>Sao Jose do Rio Preto, SP 15090-000<br>BRAZIL
1232 *	Dr. Áurea A. Paste		Dr. José R. M. de Almeida<br>Gedelias B. Nóbrega<br>Dr. Giovanna de S. Orrico<br>Mônica J. S. Silva<br>Jamile N. de Souza	Hospital Santa Izabel - Santa Casa de Misericórdia da Bahia<br>Praça Conselheiro Almeida Couto, 500<br>Nazaré<br>Salvador, BA 40050-410<br>BRAZIL	Comitê de Ética em Pesquisa Prof. Dr. Celso Figueirôa - Hospital Santa Izabel<br>Praça Conselheiro Almeida Couto, 500<br>Nazaré<br>Salvador, Bahia 40050-410<br>BRAZIL

* Did not randomize subjects

090177e1816ca80c\Approved\Approved On: 06-Oct-2010 15:16

Center	Principal Investigator	Co-Investigator(s)	Sub-Investigator(s)	Address(es)	Institutional Review Board or Ethics Committee Address(es)
1233	Edson D. Moreira Jr		Elisangela Brito Almeida<br>Debora O. R. Bonfim<br>Dr. Ricardo A. Chalhub<br>Dr. Gustavo G. V. Sampaio<br>Diego S C da Silva<br>Dr. Gustavo M. Tanajura	Associação Obras Sociais Irmã Dulce - Hospital Santo Antônio<br>Centro de Pesquisa Clínica - CPEC<br>Av Bonfim, 161<br>Largo de Roma<br>Salvador, BA 40420-000<br>BRAZIL	Comitê de Ética em Pesquisa do Hospital Santo Antônio<br>Associação Obras Sociais Irmã Dulce<br>Av. Bonfim, 161<br>Largo de Roma<br>Salvador, BA 40420-000<br>BRAZIL
1234 *	Dr. Ederlon A. C. Rezende		Raquel Q. Araujo<br>Dr. Alexandre M. Isola<br>Florencia Pereda<br>Dr. João M. Silva Junior<br>Diogo Toledo	Hospital do Servidor Público Estadual<br>Rua Pedro de Toledo, 1800 ¿ 6º andar - UTI<br>São Paulo, SP 04039-901<br>BRAZIL	CEP em Seres Humanos do Instituto de Assistência Médica ao Servidor Público Estadual<br>Rua Pedro de Toledo, 1800<br>3º andar, sala 348<br>São Paulo, SP 04039-004<br>BRAZIL
1235 *	Dr. Maria P. J. S. Lima		Michelly Baldovinotti<br>Dr. Rita C. C. Coimbra<br>Camila Z. de Deus<br>Dr. Frederico G. Palazzo<br>Dr. Raquel A. Pessagno<br>Dr. Marlirani D. C. Rocha	Hospital e Maternidade Celso Pierro da Pontifícia Universidade Católica de Campinas- PUC-Campinas<br>Av. John Boyd Dunlop, s/n<br>Jardim Ipaussurama<br>Campinas, SP 13059-900<br>BRAZIL	Comite de Ética em pesquisa com seres humanos (CEPSHP) da PUC-Campinas<br>Rua Marechal Deodoro, 1099<br>Sala 224 - Centro<br>Campinas, SP 13010-920<br>BRAZIL
1241 *	Dr. Carlos Roberto Ribeiro de Carvalho		Dr. Andre Apanavicius<br>Dr. André Luiz Dresler Hovnavian<br>Fabiane Polisel<br>Daiane de Oliveira Santos	Hospital das Clínicas da Faculdade de Medicina da Universidade de São Paulo<br>Av. Dr. Enéas de Carvalho Aguiar, 255<br>6º andar ¿ UTI Respiratoria<br>São Paulo, SP 05403-000<br>BRAZIL	Comissão de Ética para Análise de Projetos de Pesquisa CAPPesq<br>Hospital das Clínicas da Faculdade de Medicina da Universidade de São Paulo (HCFMUSP e FMUSP)<br>Rua Ovídio Pires de Campos, 225 - 5º andar<br>São Paulo, SP 05430-010<br>BRAZIL

* Did not randomize subjects

Chile

Coordinating Investigators:
<None Entered>

Center	Principal Investigator	Co-Investigator(s)	Sub-Investigator(s)	Address(es)	Institutional Review Board or Ethics Committee Address(es)
1060	Dr. Patricia Vasquez		Dr. Carmen Luz Andrade<br>Dra. Marisol Bustos<br>Dr. Pedro Miranda	Hospital San Juan De Dios<br>Huerfanos 3255<br>Santiago, RM<br>CHILE	Comite de Etica Cientifica<br>Avda. Libertador Bernardo O'Higgins 2429<br>Santiago,<br>CHILE
1061	Dr. Francisco Arancibia		Claudia Azocar<br>Javier Cerda<br>Dra. Patricia Fernández<br>Luis Soto	Instituto Nacional del Torax<br>Servicio de Medicina<br>Jose Miguel Infante 717 Piso 3°<br>Providencia, Santiago<br>CHILE	Comite Etico Cientifico<br>Servicio de Salud Metropolitano Oriente<br>Av. Salvador 364<br>Santiago,<br>CHILE
1169	Dr. Sebastian Ugarte		Cristian Grenett	Hospital del Salvador<br>UCI<br>Av. Salvador 364<br>Providencia, Santiago<br>CHILE	Comite Etico Cientifico<br>Servicio de Salud Metropolitano Oriente<br>Av. Salvador 364<br>Santiago,<br>CHILE

090177e1816ca80c\Approved\Approved On: 06-Oct-2010 15:16

Colombia

Coordinating Investigators:
<None Entered>

Center	Principal Investigator	Co-Investigator(s)	Sub-Investigator(s)	Address(es)	Institutional Review Board or Ethics Committee Address(es)
1139	Dr. Abraham Rafael Ali		Dr. Alvaro Arango	Fundacion Cardio Infantil<br>Calle 163A #28-60. 2o Piso<br>Unidad de Cuidado Intensivo<br>Bogota, Cundinamarca<br>COLOMBIA	Comite de Etica en Investigacion Clinica - Fundacion Cardio Infantil<br>Instituto de Cardiologia<br>Fundacion Cardio Infantil<br>Calle 163 A No. 28-60<br>Bogota, Cundinamarca<br>COLOMBIA
1140	Dr. Eduardo Burgos		Dr. Alirio Bastidas<br>Dr. Carlos Eduardo Perez	Hospital Militar - Unidad de Cuidado Intensivo Medica<br>Transversal 3 #49-00<br>Bogota, Cundinamarca<br>COLOMBIA	Comite de Etica en Investigacion - Hospital Militar Central<br>Hospital Militar Central<br>Transversal 3 No. 49 -00, piso 13<br>Bogota, Cundinamarca<br>COLOMBIA
1141	Dr. Guillermo Prada<br>Dr. Juan Manuel Gomez (Previous PI)		Dr. Edgar Celis	Fundacion Santa Fe de Bogota<br>Calle 119 No. 9-33<br>Bogota D.C., Cundinamarca 0<br>COLOMBIA	Comite Corporativo de Etica en Investigacion Fundacion Santa Fe de Bogota<br>Fundacion Santa Fe de Bogota<br>Calle 116 No.9 - 02<br>Bogota, Cundinamarca<br>COLOMBIA
1143	Dr. Harol Trujillo		Dr. Carlos Alberto Acosta	Hospital Federico Lleras<br>Calle 33 #4A-50 Barrio La Francia<br>Ibague, Tolima<br>COLOMBIA	Hospital Federico Lleras Acosta<br>Comite de Bioetica e Investigacion<br>Calle 33 #4a-50. Barrio La Francia<br>Ibague,<br>COLOMBIA

090177e1816ca80c\Approved\Approved On: 06-Oct-2010 15:16

Center	Principal Investigator	Co-Investigator(s)	Sub-Investigator(s)	Address(es)	Institutional Review Board or Ethics Committee Address(es)
1206	Dr. Julio C. Duran<br>Dra. Nelly E. Beltran Hoyos (Previous PI)<br>Rafael S. Cotes (Previous PI)		Dr. Jose Francisco Balaguera<br>Dra. Nelly E. Beltran Hoyos<br>Rafael S. Cotes<br>Dr. Alvaro Villanueva	Clinica de la Costa<br>Carrera 50 No. 80-90.<br>Consultorio 101<br>Barranquilla, Atlantico<br>COLOMBIA	Comite de Etica Clinica de la Costa<br>Comite de Etica<br>Clinica de la Costa<br>Carrera 50 No. 80-90<br>Barranquilla, Atlantico<br>COLOMBIA

* Did not randomize subjects

090177e1816ca80c\Approved\Approved On: 06-Oct-2010 15:16

090177e1816ca80c\Approved\Approved On: 06-Oct-2010 15:16

France

Coordinating Investigators:
<None Entered>

Center	Principal Investigator	Co-Investigator(s)	Sub-Investigator(s)	Address(es)	Institutional Review Board or Ethics Committee Address(es)
1123	Prof. Christian Auboyer		Alain Dumont<br>Arnaud Forgeot<br>Dr. Richard Jospe<br>Dr. Phillipe Mahul<br>Dr. Jerome Morel<br>Dr. David Rosay<br>Stephanie Seve<br>Raphaël Terrana	Centre Hospitalier Universitaire de Saint Etienne-Hopital Nord Service de Reanimation Polyvalente<br>118, avenue Albert Raimond<br>Saint Etienne Cedex 02, 42055<br>FRANCE	Comite de protection des personnes, Ile-de-France VI<br>Hôpital Pitié Salpêtrière<br>47 Boulevard de l'Hôpital<br>PARIS, 75013<br>FRANCE
1132	Prof. Michel Wolff		Dr. Lila Bouadma<br>Dr. Cedric Bruel	Groupe hospitalier Bichat-Claude Bernard, Service de Reanimation medicale des maladies infectieuses<br>46, rue Henri Huchard<br>Paris, Cedex 18 75877<br>FRANCE	Comite de protection des personnes, Ile-de-France VI<br>Hôpital Pitié Salpêtrière<br>47 Boulevard de l'Hôpital<br>PARIS, 75013<br>FRANCE
1133	Pr. Jean Chastre		Dr. Francois Bouvet<br>Dr. Alain Combes<br>Dr. Claude Gibert<br>Dr. Charles-Edouard Luyt<br>Dr. Mariana Mirabel<br>Dr. Ania Nieszkowska<br>Catherine Reynaud<br>Marc Tonnellier<br>Dr. Jean-louis Trouillet	Groupe Hospitalier Pitie-Salpetriere<br>Service de Reamination Medicale-Institut de Cardiologie<br>47-83 boulevard de l'Hopital<br>Paris, 75013<br>FRANCE	Comite de protection des personnes, Ile-de-France VI<br>Hôpital Pitié Salpêtrière<br>47 Boulevard de l'Hôpital<br>PARIS, 75013<br>FRANCE

* Did not randomize subjects

Center	Principal Investigator	Co-Investigator(s)	Sub-Investigator(s)	Address(es)	Institutional Review Board or Ethics Committee Address(es)
1188	Prof. Claude Denis Martin		Dr. Jacques Albanese<br>Dr. Francois Antonini<br>Dr. Valery Blasco<br>Dr. Aude Charvet<br>Frederic Garcin<br>Dr. Marc Leone<br>Dr. Pierre Visintini<br>Dr. Sandrine Wiramus<br>Dr. Laurent Zieleskiewicz	Centre Hospitalier Universitaire de Marseille, Hôpital Nord, Département d¿Anesthésie - Réanimation<br>Chemin des Bourrely<br>Marseille Cedex 20 , France 13915<br>FRANCE	Comite de protection des personnes, Ile-de-France VI<br>Hôpital Pitié Salpêtrière<br>47 Boulevard de l'Hôpital<br>PARIS, 75013<br>FRANCE

090177e1816ca80c\Approved\Approved On: 06-Oct-2010 15:16

* Did not randomize subjects

090177e1816ca80c\Approved\Approved On: 06-Oct-2010 15:16

Germany

Coordinating Investigators:
<None Entered>

Center	Principal Investigator	Co-Investigator(s)	Sub-Investigator(s)	Address(es)	Institutional Review Board or Ethics Committee Address(es)
1124	Prof. Dr. Michael Quintel		Dr. Thorsten Perl	Universitaetsklinik Goettingen<br>Abteilung Anaesthesiologie II-Operative Intensivmedizin<br>Robert-Koch-Str 40<br>Goettingen, 37075<br>GERMANY	Ethik-Kommission an der Medizinischen Fakultaet der Universitaet Leipzig<br>Institut fuer Klinische Pharmakologie<br>Haertelstrasse 16-18<br>Leipzig, 04107<br>GERMANY
1125	Dr. med. Dierk V. Schmitt		Dr. Med. Evaldas Girdauskas<br>Dr.med. Ardwan Rastan<br>Dr.med. Matthias Sauer<br>Dr. Med. Thomas Schroeter	Herzzentrum Leipzig<br>Struempellstr. 39<br>Leipzig, 04289<br>GERMANY	Ethik-Kommission an der Medizinischen Fakultaet der Universitaet Leipzig<br>Institut fuer Klinische Pharmakologie<br>Haertelstrasse 16-18<br>Leipzig, 04107<br>GERMANY
1128	Prof. Dr. med. Bernhard Ruf		Dr. Joerg Hofmann<br>Dr. Dany Plassmann<br>Dr. Till Treutler	Klinikum St. Georg<br>2. Innere Klinik, Zentrum fuer Infektiologie und Reisemedizin<br>Delitzscher Str. 141<br>Leipzig, 04129<br>GERMANY	Ethik-Kommission an der Medizinischen Fakultaet der Universitaet Leipzig<br>Institut fuer Klinische Pharmakologie<br>Haertelstrasse 16-18<br>Leipzig, 04107<br>GERMANY

Greece

Coordinating Investigators:
<None Entered>

Center	Principal Investigator	Co-Investigator(s)	Sub-Investigator(s)	Address(es)	Institutional Review Board or Ethics Committee Address(es)
1100	John Kioumis		Dr. Nikolaos Manolakoglou<br>Dr. Georgia Pitsiou	'G. Papanikolaou' District General Hospital of Thessaloniki<br>Exohi<br>Thessaloniki, 57010<br>GREECE	Georgios Papanikolaou Hospital of Thessaloniki<br>EXOHI<br>THESSALONIKI, 57010<br>GREECE
1186	Prof. Dimitris Georgopoulos		Christina Alexopoulou<br>Sevasti Koumiotaki<br>Maria Plataki<br>Katerina Vaporidi<br>Georgios Xirouchakis	University General Hospital of Heraklion<br>Voutes<br>Heraklion<br>Crete, Greece 71110<br>GREECE	PEPAGNI Hospital Heraklion Crete<br>Voutos & Stavrakion str<br>CRETE, 71110<br>GREECE
1187	Prof. George Baltopoulos		Eleni Boutzouka<br>Dr. Penelope Evagelopoulou<br>Georgios Fildissis<br>Dr. Pavlos Myrianthefs	General Hospital of Attica_KAT<br>2, Nikis Street<br>Kifisia, Athens 14561<br>GREECE	"KAT" General Hospital of Athens<br>2 Nikis str.<br>ATHENS, 14561<br>GREECE
1191	Prof. Charalabos Roussos		Dr. Spyros Kolias<br>Vassiliki Markaki<br>Dr. Christina Routsi<br>Grigorios Stratakos	General Hospital of Athens Evangelismos<br>45-47 Ipsilantou<br>Athens, 10676<br>GREECE	"Evangelismos" General Hospital of Athens<br>45-47 Ipsilantou str.<br>ATHENS, 10676<br>GREECE

* Did not randomize subjects

090177e1816ca80c\Approved\Approved On: 06-Oct-2010 15:16

Hong Kong

Coordinating Investigators:
Dr. Lee Sung Lau
Dr. Yat Yee Natalie Leung

Center	Principal Investigator	Co-Investigator(s)	Sub-Investigator(s)	Address(es)	Institutional Review Board or Ethics Committee Address(es)
1091	Dr. Wai Ming Chan		Dr. Matthew Wong<br>Dr. Karl Kang Young	The University of Hong Kong,<br>Queen Mary Hospital<br>Department of Anaesthesia and Intensive Care<br>102 Pokfulam Road<br>Hong Kong,<br>HONG KONG	Institutional Review Board of the University of Hong Kong/Hospital Authority Hong Kong West Cluster<br>Institutional Review Board of the University of Hong Kong/Hospital Authority Hong Kong West Cluster<br>Rm 901, Administration Block<br>Queen Mary Hospital<br>102 Pokfulam Road<br>Hong Kong,<br>HONG KONG
1095 *	Dr. Charles Gomersall		Prof. Gavin Joynt<br>Dr. Qi Tian<br>Dr. Li Weng	Prince of Wales Hospital<br>30-32 Ngan Shing Street<br>Shatin, New Territories<br>HONG KONG	Joint The Chinese University of H.K.-New Territories East Cluster<br>Clinical Research Ethics Committee<br>Flat 3C, Block B, Staff Quarters<br>Prince of Wales Hospital<br>Shatin,<br>HONG KONG

* Did not randomize subjects

090177e1816ca80c\Approved\Approved On: 06-Oct-2010 15:16

090177e1816ca80c\Approved\Approved On: 06-Oct-2010 15:16

Italy

Coordinating Investigators:
<None Entered>

Center	Principal Investigator	Co-Investigator(s)	Sub-Investigator(s)	Address(es)	Institutional Review Board or Ethics Committee Address(es)
1150 *	Dr. Giovanni Paolo Ligia		Elisabetta Sortino	U.O. IV Pneumologia<br>PO "R. Binaghi"<br>USL8 Sardegna<br>Via Is Guadazzonis, Cagliari 2<br>09126<br>ITALY	Comitato Etico Indipendente<br>Azienda USL 8- Cagliari<br>Via Logudoro, 17<br>Cagliari, 09127<br>ITALY
1152 *	Dr. Alberto Casazza<br>Dr. Martin Langer<br>(Previous PI)		Dr. Andrea Bottazzi<br>Dr. Federico Capra<br>Marzani<br>Dr. Vincenzo Emmi<br>Dr. Paolo Fumagalli<br>Dr. Alessandro Lilla	Anestesia e Rianimazione I e II<br>Policlinico San Matteo IRCCS<br>Via Golgi, 19<br>Pavia, 27100<br>ITALY	Comitato di Bioetica I.R.C.C.S.<br>Policlinico S.Matteo<br>V.le Golgi,19<br>pavia, 27100<br>ITALY

* Did not randomize subjects

Korea, Republic Of

Coordinating Investigators:
<None Entered>

Center	Principal Investigator	Co-Investigator(s)	Sub-Investigator(s)	Address(es)	Institutional Review Board or Ethics Committee Address(es)
1193	Yang Soo Kim		Seong-Ho Choi<br>Jae Phil Choi<br>Sang-Ho Choi<br>Yong Pill Chong<br>Eun Young Jang<br>Min Hyok Jeon<br>Jung Im Kim<br>Dr. Mi-Na Kim<br>Min Kyung Kim<br>Seong Cheol Kim<br>Tae Kyung Kwon<br>Sang-Oh Lee<br>Ki-Ho Park<br>Kyung Ah Song	Asan Medical Center, Division of Infectious Disease<br>388-1, Pungnap-dong, Songpa-gu<br>Seoul, 138-736<br>KOREA, REPUBLIC OF	IRB of Asan Medical Center<br>Asan Medical Center IRB<br>388-1 Pungnap-dong, Songpa-gu<br>Seoul, 138-736<br>KOREA, REPUBLIC OF
1194	Joong-Sik Eom		Kyoung Woo Dong<br>Bo-Young Hwang<br>Yang Hwa Kim<br>Sun-young Kwon<br>Ms. Young-Jin Lee<br>Jin Seo Lee<br>Eun Kyung Mo<br>Yong-Bum Park<br>So-Yeon Park<br>Sul-Hee Sim	Kangdong Sacred Heart Hospital, Hallym University Medical Center, Division of Infectious Diseases<br>445 Gil-Dong, Kangdong-Gu<br>Seoul, 134-701<br>KOREA, REPUBLIC OF	IRB of Kangdong Sacred Heart Hospital, Hallym University Medical Center<br>IRB of Kangdong Sacred Heart Hospital, Hallym University Medical Center<br>445 Gil-dong, Kangdong-gu<br>Seoul, 134-701<br>KOREA, REPUBLIC OF

090177e1816ca80c\Approved\Approved On: 06-Oct-2010 15:16

Center	Principal Investigator	Co-Investigator(s)	Sub-Investigator(s)	Address(es)	Institutional Review Board or Ethics Committee Address(es)
1195	Prof. Min Ja Kim		Sun Hwa Chung<br>Dr. Sung-Bum Kim<br>Dr. Jeong-Yeon Kim<br>JU HEUI KIM<br>Sung hee Park<br>Dr. Yoonseon Park<br>Yeon Doo Ryu<br>Dr. Jang-Wook Sohn<br>Dr. Young Kyung Yoon	Korea University Anam Hospital,<br>Division of Infectious Diseases<br>126-1, 5th, Anam-Dong,<br>Seongbuk-Gu<br>Seoul, 136-705<br>KOREA, REPUBLIC OF	IRB of Korea University Anam Hospital<br>IRB of Korea University Anam Hospital<br>126-1, 5th, Anam-Dong, Seongbuk-Gu<br>Seoul, 136-705<br>KOREA, REPUBLIC OF
1222	Prof. Kyong Ran Peck		Dr. HaeSuk Cheong<br>Kwon Gyeong Won<br>Young Eun Ha<br>Dr. Mi-Kyong Joung<br>Shin Han Jung<br>Dr. EunSeok Kim<br>Gyeong Won Kwon<br>Prof. Nam Yong Lee<br>Kang Mingyoung<br>Hye Won Moon<br>Kyung Mok Sohn<br>Dr. Gee Young Suh	Samsung Medical Center,<br>Division of Infectious Diseases<br>50 Ilwon-dong Kangnam-gu<br>Seoul, 135-710<br>KOREA, REPUBLIC OF	Samsung Medical Center IRB<br>Samsung Medical Center<br>50 Ilwon-dong, Kangnam-Gu<br>Seoul, 135-710<br>KOREA, REPUBLIC OF
1223	Prof. Dong-Gun Lee<br>Prof. Wan-shik Shin<br>(Previous PI)		Prof. Su-mi Choi<br>Hye-Rim Jung<br>Dr. Sae Yoon Kee<br>Dr. Si-Hyun Kim<br>Hyun-Ji Kim<br>Hyun-Jin Kim<br>Yo A Lee	Division of Infectious Disease,<br>The Catholic University of<br>Korea, St. Mary's Hospital<br>62, Yeouido-dong,<br>Yeongdeungpo-gu<br>Seoul, 150-713<br>KOREA, REPUBLIC OF	St. Mary's hospital IRB<br>St. Mary's hospital IRB<br>62, Yeouido-dong ,Yeongdeungpo-gu<br>Seoul, 150-713<br>KOREA, REPUBLIC OF

090177e1816ca80c\Approved\Approved On: 06-Oct-2010 15:16

Malaysia

Coordinating Investigators:
<None Entered>

Center	Principal Investigator	Co-Investigator(s)	Sub-Investigator(s)	Address(es)	Institutional Review Board or Ethics Committee Address(es)
1092	Prof. Adeeba Kamarulzaman		Dr. Norliza Ariffin<br>Dr Ai-Vyrn Chin<br>Dr. Shahrul Bahyah Kamaruzzaman<br>Dr. Kwee-choy James Koh<br>Dr. Soraya Kunanayagam<br>Assoc. Prof. Dr. Yong Kek Pang<br>Dr. Sasheela Sri La Sri Ponnampalavanar<br>Dr. Sharifah Faridah Syed Omar	University Malaya Medical Centre<br>Lembah Pantai<br>Kuala Lumpur, 59100<br>MALAYSIA	Medical Ethics Committee<br>University Malaya Medical Centre<br>Lembah Pantai<br>Kuala Lumpur, 59100<br>MALAYSIA
1101	Dr. Li Ling Tai		Dr. Siew Hian Ng<br>Dr. Shanti Rudra Deva	Hospital Kuala Lumpur<br>Jalan Pahang<br>Kuala Lumpur, 50586<br>MALAYSIA	Medical Research & Ethics Committee<br>Ministry of Health, c/o NIH<br>Secretariat, Institute for Health Management<br>Jalan Rumah Sakit<br>Bangsar<br>Kuala Lumpur, 59000<br>MALAYSIA

090177e1816ca80c\Approved\Approved On: 06-Oct-2010 15:16

090177e1816ca80c\Approved\Approved On: 06-Oct-2010 15:16

Mexico

Coordinating Investigators:
Magaly Flores-Ortiz (Previous Coordinating Investigator)

Center	Principal Investigator	Co-Investigator(s)	Sub-Investigator(s)	Address(es)	Institutional Review Board or Ethics Committee Address(es)
1080	Dr. Eduardo Rodriguez-Noriega		Dr. Sayra Camarena<br>Dr. Pedro Gomez Quiroz<br>Dr. Rayo Morfin-Otero<br>Dr. Marisela Vazquez Leon	Hospital Civil De Guadalajara<br>Calle Hospital 308, Col. Centro<br>Guadalajara, Jalisco 44280<br>MEXICO	Comite de Enseñanza, Investigacion y Etica<br>Calle Hospital 278<br>Sector Hidalgo<br>Guadalajara, Jalisco 44280<br>MEXICO
1081	Dr. Cesar Cruz-Lozano		Dr. Fernando Corona-Fernandez<br>Jorge Rosendo Sanchez-Medina	Hospital Regional de Petróleos Mexicanos<br>Calle 10 y 5a. Avenida<br>Col. Jardin 20 de Noviembre<br>Ciudad Madero, Tamaulipas 89440<br>MEXICO	Hospital Regional de Petroleos Mexicano<br>Comité de Investigación y Bioetica<br>Calle 10 y 5a. Avenida Col. Jardin 20 de Noviembre<br>Ciudad Madero, Tamaulipas 89440<br>MEXICO
1082	Dr. Luis Adrian Rendon-Perez		Dr. Hilda Alfaro-Riveros<br>Dr. Janeth A. Almaguer<br>Dr. Sandra-Leticia Cervantes-de-Hoyos<br>Paulina Cruz-Bravo<br>Dr. Abril Deschamps-Blanco<br>Mariana Rodriguez-Leal<br>Farah Katia Sevilla<br>Letycia Alejandrina Zavala-Gomez	Hospital Universitario Dr. José E. Gonzalez Edificio Rodrigo Barragan 3o Piso<br>C.I.P.T.I.R. Centro de investigación Prevención y Tratamiento de Infecciones Respiratórias<br>Av. Madero y Gonzalitos S/N<br>Monterrey/Col. Mitras Centro, Nuevo Léon 64460<br>MEXICO	Comité de Investigación y Ética<br>Hospital Universitario Dr. José E. Gonzalez<br>Madero y Gonzalitos s/n<br>Col. Mitras Centro<br>Monterrey, Nuevo León 64460<br>MEXICO

* Did not randomize subjects

Center	Principal Investigator	Co-Investigator(s)	Sub-Investigator(s)	Address(es)	Institutional Review Board or Ethics Committee Address(es)
1155	Dr. Guillermo M. Ruiz Palacios		Dr. Pablo Belaunzaran-Zamudio<br>Dr. Claudia Mendez-Mercado	Instituto Nacional de Ciencias Medicas y Nutrición "Salvador Zubiran".Departamento de Infectologia<br>Vasco de Quiroga #15<br>Col. Seccion XVI. Delegacion Tlalpan<br>Mexico, DF 14000<br>MEXICO	Instituto Nacional de Ciencias Medicas y Nutricion Salvador Zubiran<br>Comite Institucional de Investigacion Biomedica en Humanos<br>VASCO DE QUIROGA 15<br>COL SECCION XVI TLALPAN<br>Mexico, DF 14000<br>MEXICO

090177e1816ca80c\Approved\Approved On: 06-Oct-2010 15:16

090177e1816ca80c\Approved\Approved On: 06-Oct-2010 15:16

Poland

Coordinating Investigators:
<None Entered>

Center	Principal Investigator	Co-Investigator(s)	Sub-Investigator(s)	Address(es)	Institutional Review Board or Ethics Committee Address(es)
1212	Prof. Barbara Zubelewicz-Szkodzinska		Dr. Aleksander Danikiewicz<br>Dr. Lukasz Labus<br>Dr. Anna Pietka-Rzycka<br>Dr. Wojciech Romanowski	Szpital Specjalistyczny nr 1<br>Katedra i Klinika Chorob Wewnetrznych w Bytomiu<br>ul. Zeromskiego 7<br>Bytom, 41-902<br>POLAND	Komisja Bioetyczna Slaskiego Uniwersytetu Medycznego w Katowicach<br>Poniatowskiego 15<br>Katowice, 40-055<br>POLAND
1213	Prof. Wladyslaw Pierzchala		Dr. Grzegorz Gasior<br>Dr. Mariola Ograbek-Krol<br>Dr. Anna Oslawska-Dzierzega	Samodzielny Publiczny Centralny Szpital Kliniczny im. Prof. Kornela Gibinskiego Slaskiego Uniwersytetu Medycznego w Katowicach, Klinika Pneumonologii<br>ul. Medykow 14<br>Katowice, 40-752<br>POLAND	Komisja Bioetyczna Slaskiego Uniwersytetu Medycznego w Katowicach<br>Poniatowskiego 15<br>Katowice, 40-055<br>POLAND

* Did not randomize subjects

Center	Principal Investigator	Co-Investigator(s)	Sub-Investigator(s)	Address(es)	Institutional Review Board or Ethics Committee Address(es)
1226	Prof. Andrzej Szczeklik		Dr. Stanislawa Bazan - Socha<br>Dr. Aleksandra Bukiej<br>Dr. Joanna Marciniak-Sroka<br>Dr. Lucyna Mastalerz<br>Dr. Wojciech Wegrzyn	SPZOZ Szpital Uniwersytecki w Krakowie<br>Oddzial Kliniczny Kliniki Alergii i Immunologii<br>ul. Skawinska 8<br>Krakow, 31 - 066<br>POLAND<br><br>SPZOZ Szpital Uniwersytecki w Krakowie<br>Oddzial Autoimmunologii i Zaburzen Hemostazy<br>ul. Skawinska 8<br>Krakow, 31-066<br>POLAND<br><br>SPZOZ Szpital Uniwersytecki w Krakowie<br>Oddzial Pulmonologii<br>ul. Skawinska 8<br>Krakow, 31-066<br>POLAND	Komisja Bioetyczna Slaskiego Uniwersytetu Medycznego w Katowicach<br>Poniatowskiego 15<br>Katowice, 40-055<br>POLAND

090177e1816ca80c\Approved\Approved On: 06-Oct-2010 15:16

* Did not randomize subjects

Portugal

Coordinating Investigators:
<None Entered>

Center	Principal Investigator	Co-Investigator(s)	Sub-Investigator(s)	Address(es)	Institutional Review Board or Ethics Committee Address(es)
1121	Dra. Paula Coutinho		Dra. Regina Coelho	Centro Hospitalar de Coimbra/Unidade de Cuidados Intensivos<br>Quinta dos Vales-S. Martinho do Bispo<br>Coimbra, 3041<br>PORTUGAL	CEIC - Comissão de Ética para a Investigação Clínica<br>Parque da Saúde de Lisboa - Av[a] do Brasil, 53- Pavilhão 17-A<br>Lisboa, 1749-004<br>PORTUGAL<br><br>Comissão de Ética do Centro Hospitalar de Coimbra<br>Centro Hospitalar de Coimbra<br>S. Martinho do Bispo<br>Coimbra, 3040<br>PORTUGAL
1122	Dr. Eduardo Jorge Almeida		Dr. Amanda Alves<br>Dr. Antero do Vale Fernandes<br>Dra. Sara Beatriz Lança<br>Dra. Lucinda Oliveira	Unidade de Cuidados Intensivos<br>Hospital Garcia de Orta<br>Bairro do Matadouro<br>Pragal<br>Almada, 2800<br>PORTUGAL	CEIC - Comissão de Ética para a Investigação Clínica<br>Parque da Saúde de Lisboa - Av[a] do Brasil, 53- Pavilhão 17-A<br>Lisboa, 1749-004<br>PORTUGAL<br><br>Comissão de Ética - Hospital Garcia de Orta<br>Comissão de Ética - Hospital Garcia de Orta<br>Bairro do Matadouro<br>Almada, 2825<br>PORTUGAL

090177e1816ca80c\Approved\Approved On: 06-Oct-2010 15:16

Center	Principal Investigator	Co-Investigator(s)	Sub-Investigator(s)	Address(es)	Institutional Review Board or Ethics Committee Address(es)
1130	Dra. Anabela Mesquita		Dr. Pedro Grade	Unidade de Saude Local de Matosinhos/Hospital Pedro Hispano-Unidade de Cuidados Intensivos<br>Rua Dr. Eduardo Torres<br>Senhora da Hora, 4464-513<br>PORTUGAL	CEIC - Comissão de Ética para a Investigação Clínica<br>Parque da Saúde de Lisboa - Avª do Brasil, 53- Pavilhão 17-A<br>Lisboa, 1749-004<br>PORTUGAL<br><br>Comissão de Ética da Unidade Local de Saúde de Matosinhos SA<br>Unidade Local de Saúde de Matosinhos SA<br>Matosinhos,<br>PORTUGAL
1173	Dr. Pedro Ferreira (Previous PI)<br>Dra Camila Tapadinhas			Hospital S. Francisco Xavier-Servico Medicina- Unidade de Cuidados Intensivos<br>Estrada do Forte do Alto do Duque<br>Lisboa, 1449-005<br>PORTUGAL	CEIC - Comissão de Ética para a Investigação Clínica<br>Parque da Saúde de Lisboa - Avª do Brasil, 53- Pavilhão 17-A<br>Lisboa, 1749-004<br>PORTUGAL<br><br>Comissão de Ética - Hospital S. Francisco Xavier<br>Comissão de Ética - Hospital S. Francisco Xavier<br>Estrada do Forte do Alto do Duque<br>Lisboa, 1449-005<br>PORTUGAL

090177e1816ca80c\Approved\Approved On: 06-Oct-2010 15:16

090177e1816ca80c\Approved\Approved On: 06-Oct-2010 15:16

Russian Federation

Coordinating Investigators:
<None Entered>

Center	Principal Investigator	Co-Investigator(s)	Sub-Investigator(s)	Address(es)	Institutional Review Board or Ethics Committee Address(es)
1105	Prof. Boris R. Gelfand		Dr. Elizaveta B. Gelfand<br>Dr. Alexander N. Martynov	Dept. of Surgery,Anasthesiology and Intencive Care<br>8, Leninsky Prospect<br>Moscow, RUSSIA 117049<br>RUSSIAN FEDERATION	Ethicheskii Comitet Gosudarstvennogo Obrazovatelnogo Uchrezhdeniya RGMU<br>1. UL. Ostrovitlanova<br>Moskva, 117997<br>RUSSIAN FEDERATION<br><br>Ethicheskii Comitet Pri Federalnom Organe Kontrolia Kachestva Lekarstvennikh Sredstv<br><br>Petrovskii Boulvar 8, Build. 3<br>Moskva, 127051<br>RUSSIAN FEDERATION
1106	Prof. Sergey V. Yakovlev		Dr. Irina N. Bouseva<br>Dr. Yuriy Y. Romanovskiy<br>Dr. Oleg M. Romashov<br>Dr. Tatiana V. Shakhova	Gosudarstvennoe Uchrezhdenie Zdravookhraneniya goroda Moskvy Gorodskaya Klinicheskaya Bolnitsa #7 Komiteta Zdravookhraneniya Moskvy<br>Otdelenie reanimacii i intensivnoy terapii<br>4, Kolomenskiy Proezd<br>Moscow, 115446<br>RUSSIAN FEDERATION	Ethicheskiy Comitet pri Gosudarstvennom Uchrezhdenii Zdravookhraneniya goroda Moskvy Gorodskaya Klinicheskaya Bolnitsa #7 Komiteta Zdravookhraneniya Moskvy<br>4, Kolomenskiy Proezd<br>Moscow, 115446<br>RUSSIAN FEDERATION<br><br>Ethics Committee at the Federal Service on Surveillance in Healthcare and Social Development<br>8, str. 2, Petrovskij bulvar<br>Moscow, 127051<br>RUSSIAN FEDERATION

* Did not randomize subjects

Center	Principal Investigator	Co-Investigator(s)	Sub-Investigator(s)	Address(es)	Institutional Review Board or Ethics Committee Address(es)
1117	Dr. Irina E. Gridchik		Dr. Galina G. Borisova<br>Dr. Alla V. Khlyustina<br>Dr. Anna A. Rog	Gosudarstvennoe Uchrezhdenie Zdravookhraneniya goroda Moskvy Gorodskaya Klinicheskaya Bolnitsa #15 im. O. M. Filatova Komiteta Zdravookhraneniya g. Moskvy Otdelenie Intensivnoy Terapii<br>23 Veshnyakovskaya ul.<br>Moscow, 111539<br>RUSSIAN FEDERATION	Ethicheskiy Comitet Pri Gosudarstvennoe Uchrezhdenie Zdravookhraneniya goroda Moskvy Gorodskaya Klinicheskaya Bolnitsa # 15 im. O.M. Filatova Komiteta Zdravookhraneniya g. Moskvy<br>23 Veshnyakovskaya ul.<br>Moscow, 111539<br>RUSSIAN FEDERATION<br><br>Ethics Committee at the Federal Service on Surveillance in Healthcare and Social Development<br>8, str. 2, Petrovskij bulvar<br>Moscow, 127051<br>RUSSIAN FEDERATION

* Did not randomize subjects

090177e1816ca80c\Approved\Approved On: 06-Oct-2010 15:16

Center	Principal Investigator	Co-Investigator(s)	Sub-Investigator(s)	Address(es)	Institutional Review Board or Ethics Committee Address(es)
1118	Prof. Grigory P. Aroutyunov		Dr. Liana T. Alikhanova<br>Dr. Natalia V. Rylova<br>Dr. Anna Rylova<br>Dr. Eugeniy Y. Smoltsov	City Hospital #4, Department of Therapy of Moscow Faculty of Russian State Medical University<br>Pavlovskaya str., 25, building 13<br>Moscow, 113093<br>RUSSIAN FEDERATION<br><br>Kafedra Terapii Moskovskogo Fakulteta Gosudarstvennogo Obrazovatelnogo Uchrezhdeniya Vysshego Professionalnogo Obrazovaniya Rossiyskogo Gosudarstvennogo Meditsinskogo Universiteta Gosudarstvennoc Uchrezhdenie Zdravookhraneniya goroda Moskvy Gorodskaya Klinicheskaya Bolnitsa #4 Departamenta Zdravookhraneniya goroda Moskvy/ 25, ul, Pavlovskaya<br>Moskva, 115093<br>RUSSIAN FEDERATION	Ethicheskii Comitet Gosudarstvennogo Obrazovatelnogo Uchrezhdeniya RGMU<br>1. UL. Ostrovitlanova<br>Moskva, 117997<br>RUSSIAN FEDERATION<br><br>Ethicheskii Comitet Pri Federalnom Organe Kontrolia Kachestva Lekarstvennikh Sredstv<br><br>Petrovskii Boulvar 8, Build. 3<br>Moskva, 127051<br>RUSSIAN FEDERATION
1119	Prof. Nikolai M.J. Fedorovskiy		Dr. Sergey Y. Bastrikin<br>Dr. Alexey M. Ovechkin<br>Dr. Alexander A. Petrov	Gosudarstvennoe Uchrezhdenie Zdravookhraneniya Goroda Moskvy Gorodskaya Klinicheskaya Bolnitsa #67 Komiteta Zdravookhraneniya Moskvy, Otdelenie Intensivnoy Therapii<br>UL. Salyama Adilya, D.2<br>Moscow, 123448<br>RUSSIAN FEDERATION	Ethicheskii Comitet Pri Federalnom Organe Kontrolia Kachestva Lekarstvennikh Sredstv<br><br>Petrovskii Boulvar 8, Build. 3<br>Moskva, 127051<br>RUSSIAN FEDERATION<br><br>Ethicheskii Comitet pri Gorodskaya klinicheskaya bolnitsa # 67<br>ul. Salyama Adilya,d.2<br>Moskva, 123448<br>RUSSIAN FEDERATION

090177e1816ca80c\Approved\Approved On: 06-Oct-2010 15:16

Center	Principal Investigator	Co-Investigator(s)	Sub-Investigator(s)	Address(es)	Institutional Review Board or Ethics Committee Address(es)
1225	Dr. Natalia V. Dmitrieva		Dr. Zlata V. Grigoryevskaya<br>Dr. Elena Valentinovna Kulaga<br>Dr. Irina N. Petukhova<br>Dr. Elena Nikolayevna Sokolova	National Cancer Research Center RAMS; Laboratory Microbiological Diagnostics<br>Kashirskoe shosse, 24,<br>Moscow, 115478<br>RUSSIAN FEDERATION	Ethics Committee at the Federal Service on Surveillance in Healthcare and Social Development<br>8, str. 2, Petrovskij bulvar<br>Moscow, 127051<br>RUSSIAN FEDERATION<br><br>Ethics Committee of RUSSIAN ONCOLOGICAL RESEARCH CENTER n.a. N.N. BLOKHIN RAMS<br>Kashirskoe shosse, 24<br>Moscow, 115478<br>RUSSIAN FEDERATION

090177e1816ca80c\Approved\Approved On: 06-Oct-2010 15:16

* Did not randomize subjects

Singapore

Coordinating Investigators:
<None Entered>

Center	Principal Investigator	Co-Investigator(s)	Sub-Investigator(s)	Address(es)	Institutional Review Board or Ethics Committee Address(es)
1098	Dr. Jagadesan Raghuram (Previous PI)<br>Dr. Kenneth Ping Wah Chan		Dr. Constance Pau Lin Lo<br>Dr. Ghee Chee Phua<br>Dr. Steve Tze Yi Yang	Singapore General Hospital<br>Outram Road<br>,<br>SINGAPORE	SingHealth Centralized Institutional Review Board<br>Singapore Health Services Pte Ltd<br>Blk A, 7 Hospital Drive,<br>SingHealth Research Facilities, #03-01<br>Singapore, Singapore 169611<br>SINGAPORE

090177e1816ca80c\Approved\Approved On: 06-Oct-2010 15:16

South Africa

Coordinating Investigators:
<None Entered>

Center	Principal Investigator	Co-Investigator(s)	Sub-Investigator(s)	Address(es)	Institutional Review Board or Ethics Committee Address(es)
1108	Dr. Murimisi Demmy Mukansi		Dr. John O. Opolot	Helen Joseph Hospital<br>Perth Road<br>Auckland Park, 2006<br>SOUTH AFRICA	University of the Witwatersrand<br>Human Research Ethics Committee(Medical)<br>Division of the Deputy Registrar (Research)<br>Department of Research, Senate House<br>University of the Witwatersrand<br>1 Jan Smuts Avenue<br>Braamfontein, Johannesburg 2000<br>SOUTH AFRICA
1109	Dr. Brandon William Piketh		Dr. Christine Banage<br>Dr. Jacqueline Monika Brown<br>Dr. Linda Gabrielle Doedens<br>Dr. Lufuno Rudo Mathivha<br>Dr. Shahed Omar<br>Dr. Remy M'Pio Toko<br>Dr. Richard Peter Von Rahden	Chris Hani Baragwanath Hospital<br>Old Potchefstroom Road<br>Soweto, 2013<br>SOUTH AFRICA	University of Witwatersrand Human Ethics Committee (Medical)<br>Division of the Deputy Registrar (Research), Department of Research, Senate House<br>University of the Witwatersrand<br>1 Jan Smuts Avenue<br>Braamfontein, Johannesburg 2000<br>SOUTH AFRICA
1113 *	Dr. Justus G. Kilian		Dr Joachim Hendrik Venter	Wilgers Hospital<br>Denneboom Avenue<br>Die Wilgers X14, 0041<br>SOUTH AFRICA<br><br>Wilmed Medical Research Projects<br>28 Beuke Place<br>Die Wilgers X 14, 0041<br>SOUTH AFRICA	Pharma - Ethics Independent Research Ethics Committee<br>123 Amcor Road<br>Lyttleton Manor, 0157<br>SOUTH AFRICA

* Did not randomize subjects

090177e1816ca80c\Approved\Approved On: 06-Oct-2010 15:16

Center	Principal Investigator	Co-Investigator(s)	Sub-Investigator(s)	Address(es)	Institutional Review Board or Ethics Committee Address(es)
1114	Dr. Gilbert John Gibson		Dr. Frederik Christoffel Johannes Bester<br>Ignatius P. Immink	Rose Park Hospital<br>53 Gustav Crescent<br>Bloefontein, 9301<br>SOUTH AFRICA	Pharma - Ethics Independent Research Ethics Committee<br>123 Amcor Road<br>Lyttleton Manor, 0157<br>SOUTH AFRICA

090177e1816ca80c\Approved\Approved On: 06-Oct-2010 15:16

* Did not randomize subjects

Spain

Coordinating Investigators:
<None Entered>

Center	Principal Investigator	Co-Investigator(s)	Sub-Investigator(s)	Address(es)	Institutional Review Board or Ethics Committee Address(es)
1103	Dr. Manuel Soler		Hipolito E. Perez Molto<br>JAVIER SARMIENTO	HOSPITAL DE BADALONA GERMANS TRIAS I PUJOL<br>SERVICIO DE UCI<br>CARRETERA DEL CANYET, S/N<br>BADALONA, BARCELONA 08916<br>SPAIN	Hospital Universitari Germans Trias i Pujol<br>Ethics Committee of Clinic Investigation<br>Ctra del Canyet, s/n<br>Servicio de Farmacologia Clinica<br>Badalona, Barcelona 08916<br>SPAIN
1104 *	Dr. Emilio S. Bouza		Juan Manuel Garcia<br>Paloma Gijon<br>Dr. Patricia Munoz<br>Belen Padilla<br>Mar Sanchez	HOSPITAL UNIVERSITARIO GREGORIO MARAÑON<br>SERVICIO DE MICROBIOLOGIA<br>C/ DR. ESQUERDO, 46<br>MADRID, MADRID 28009<br>SPAIN	Hospital General Universitario Gregorio Marañon<br>Ethics Committee of Clinic Investigation<br>C/ Dr. Esquerdo, Nº 46<br>Madrid, 28007<br>SPAIN
1115	Dr. Francisco Alvarez Lerma		Yolanda Diaz Buendia<br>Dr. Dolores Marzo<br>Ana Isabel Perez Martin	Hospital Del Mar/Servicio de medicina intensiva<br>Paseo Maritimo 25-29<br>Barcelona, Barcelona 08003<br>SPAIN	Comite Etico de Investigacion Clinica<br>Instituto Municipal de Asistencia Sanitaria (IMAS)<br>Calle Doctor Aiguader, no. 80<br>Barcelona, 08003<br>SPAIN

* Did not randomize subjects

090177e1816ca80c\Approved\Approved On: 06-Oct-2010 15:16

090177e1816ca80c\Approved\Approved On: 06-Oct-2010 15:16

Center	Principal Investigator	Co-Investigator(s)	Sub-Investigator(s)	Address(es)	Institutional Review Board or Ethics Committee Address(es)
1116	Dr. Antonio Torres		Silvia Blanco<br>Dr. Miguel Ferrer<br>Adamantia Liapikou<br>Dr. Antonio Moreno<br>Dr. Mauricio Valencia	Hospital Clinic I Provincial<br>Servicio de Neumologia<br>C/ Villarroel 170<br>Barcelona, Barcelona 08036<br>SPAIN	COMITE ETICO DE INVESTIGACION CLINICA HOSPITAL CLINIC i PROVINCIAL<br>C/ VILLARROEL, 170<br>BARCELONA, 08036<br>SPAIN<br><br>Comite Etico de Investigacion Clinica (CEIC)<br>Hospital Universitario Germans trias i Pujol<br>Carretera de Canyet s/n<br>Badalona, 08916<br>SPAIN
1159	Juan Jose Picazo		Mercedes Nieto<br>Prof. Elisa Perez-Cecilia	HOSPITAL CLINICO SAN CARLOS<br>SERVICIO DE MICROBIOLOGIA<br>AVDA. PROF. MARTIN LAGOS S/N<br>MADRID, MADRID 28040<br>SPAIN	Hospital Clinico San Carlos<br>ETHICS COMMITTEE OF CLINICAL INVESTIGATION<br>C/ DR. MARTIN LAGOS, S/N<br>MADRID, MADRID 28040<br>SPAIN

Taiwan

Coordinating Investigators:
<None Entered>

Center	Principal Investigator	Co-Investigator(s)	Sub-Investigator(s)	Address(es)	Institutional Review Board or Ethics Committee Address(es)
1093	Dr. Jen-hsien Wang		Dr. Chih-Ming Chen<br>Dr. Cheng-Mao Ho	China Medical University Hospital<br>2 Yuh Der Road<br>Taichung, 404<br>TAIWAN	Research Ethics Review Committee, China Medical University Hospital<br>2 Yuh Der Road<br>Taichung, 404<br>TAIWAN
1094	Dr. Po-ren Hsueh		Dr. Kuan-Yu Chen	National Taiwan University Hospital<br>7, Chung-Shan South Road<br>Taipei, 100<br>TAIWAN	National Taiwan University Hospital, Ethics Committee<br>7 Chung Shan South Road<br>Taipei, 10012<br>TAIWAN
1096	Dr. Yao-Shen Chen		Dr. Ming-Hsin Mai<br>Dr. Yung-Hsing Wang	Kaosiung Veterans General Hospital<br>386, Ta-Chung First Road<br>Kaohsiung, 813<br>TAIWAN	Joint Institutional Review Board<br>201 Shih-Pai Road, Section 2<br>Taipei, 112<br>TAIWAN<br><br>Kaohsiung Veterans General Hospital, Institutional Review Board<br>386 Ta-Chung First Road<br>Kaohsiung, 813<br>TAIWAN
1242	Dr. Chun-Hsing Liao		Dr. Hou-Tai Chang	Far-Eastern Memorial Hospital<br>21, Nan-Ya South Road, Section 2<br>Pan-Chiao, 220<br>TAIWAN	Far Eastern Memorial Hospital, Research Ethics Review Committee<br>21, Nan-Ya South Road, Section 2<br>Pan-Chiao, Taipei<br>TAIWAN

090177e1816ca80c\Approved\Approved On: 06-Oct-2010 15:16

Turkey

Coordinating Investigators:
<None Entered>

Center	Principal Investigator	Co-Investigator(s)	Sub-Investigator(s)	Address(es)	Institutional Review Board or Ethics Committee Address(es)
1209	Prof. Dr. Serhat Unal		Dr. Defne Altintas<br>Dr. Nursel Calik Basaran<br>Assist. Prof. Dr. Kutay Demirkan<br>Arife Ozveren<br>Assoc. Prof. Dr. Arzu Topeli Iskit	Hacettepe University Medical Faculty<br>Infectious Diseases<br>Sihhiye<br>Ankara, 06100<br>TURKEY	Hacettepe University<br>Local Ethics Committee<br>Ankara,<br>TURKEY<br><br>Turkish Ministry of Health Central Ethics Committee<br>T.R. Ministry of Health<br>Pharmaceutical General Directorate<br>Central Ethics Committee /<br>Regulatory Authority<br>Cankiri Cad. 57<br>Diskapi ¿ Ulus<br>ANKARA, 06060<br>TURKEY
1214 *	Prof. Dr. Recep Ozturk		Prof. Dr. Yalim Dikmen<br>Dr. Bilgul Mete<br>Assoc. Prof. Dr. Resat Ozaras	Istanbul University Cerrahpasa Med. Fac. Clinical Microbiology and Infectious Diseases Dept.<br>Aksaray, Istanbul<br>TURKEY	Turkish Ministry of Health Central Ethics Committee<br>T.R. Ministry of Health<br>Pharmaceutical General Directorate<br>Central Ethics Committee /<br>Regulatory Authority<br>Cankiri Cad. 57<br>Diskapi ¿ Ulus<br>ANKARA, 06060<br>TURKEY<br><br>University of Istanbul<br>Local Ethics Committee Cerrahpasa Medical Faculty<br>Istanbul,<br>TURKEY

* Did not randomize subjects

090177e1816ca80c\Approved\Approved On: 06-Oct-2010 15:16

Center	Principal Investigator	Co-Investigator(s)	Sub-Investigator(s)	Address(es)	Institutional Review Board or Ethics Committee Address(es)
1215 *	Prof. Dr. Volkan Korten		Prof. Dr. Turgay Celikel<br>Dr. Emel Eryuksel<br>Dr. Lutfiye Mulazimoglu<br>Assoc. Prof. Dr. Zekaver Odabasi	Marmara University Med. Fac.<br>Infectious Diseases and Clinical Microbiology Dept.<br>Altunizade, Istanbul<br>TURKEY	Marmara University School of Med.<br>Ethics Committee<br>Istanbul,<br>TURKEY<br><br>Turkish Ministry of Health Central Ethics Committee<br>T.R. Ministry of Health<br>Pharmaceutical General Directorate<br>Central Ethics Committee /<br>Regulatory Authority<br>Cankiri Cad. 57<br>Diskapi ¿ Ulus<br>ANKARA, 06060<br>TURKEY
1216 *	Prof. Dr. Sercan Ulusoy		Dr. Bilgin Arda<br>Assoc. Prof. Dr. Feza Bacakoglu<br>Dr. Husnu Pullukcu<br>Assoc. Prof. Dr. Alper Tunger	Ege University Med. Fac.<br>Infectious Diseases and Clinical Microbiology Dept.<br>Bornova, Izmir<br>TURKEY	Ethics Committee of Medical Faculty, Ege University<br>Bornova-Izmir, 35100<br>TURKEY<br><br>Turkish Ministry of Health Central Ethics Committee<br>T.R. Ministry of Health<br>Pharmaceutical General Directorate<br>Central Ethics Committee /<br>Regulatory Authority<br>Cankiri Cad. 57<br>Diskapi ¿ Ulus<br>ANKARA, 06060<br>TURKEY

* Did not randomize subjects

090177e1816ca80c\Approved\Approved On: 06-Oct-2010 15:16

Center	Principal Investigator	Co-Investigator(s)	Sub-Investigator(s)	Address(es)	Institutional Review Board or Ethics Committee Address(es)
1217 *	Prof. Dr. Halis Akalin		Assoc. Prof. Dr. Emel Yilmaz	Uludag University Med. Fac. Microbiology and Infectious Diseases Dept.<br>Gorukle, Bursa<br>TURKEY	Turkish Ministry of Health Central Ethics Committee<br>T.R. Ministry of Health Pharmaceutical General Directorate Central Ethics Committee / Regulatory Authority<br>Cankiri Cad. 57<br>Diskapi ¿ Ulus<br>ANKARA, 06060<br>TURKEY<br><br>Uludag University School of Med. Ethics Committee<br>Tip Fakultesi Dekanligi<br>Bursa,<br>TURKEY

090177e1816ca80c\Approved\Approved On: 06-Oct-2010 15:16

United Kingdom

Coordinating Investigators:
<None Entered>

Center	Principal Investigator	Co-Investigator(s)	Sub-Investigator(s)	Address(es)	Institutional Review Board or Ethics Committee Address(es)
1126 *	Dr. Mark Wilcox			Leeds General Infirmary/ Old Medical School/ Department of Microbiology<br>Great George Street<br>Leeds, LS1 3EX<br>UNITED KINGDOM	West Midlands Research Ethics Commitee<br>West Midlands Research Ethics Commitee<br>Prospect House<br>Fishing Line Road<br>Enfield, Redditch B97 6EW<br>UNITED KINGDOM
1189	Dr. David G. Swann		Dr. Andrew Conway Morris<br>Dr. Brian Cook<br>Dr. Nazir Lone<br>Dr. Olga Lucia Moncayo<br>Dr. Timothy Simon Walsh	Royal Infirmary of Edinburgh<br>Dept. of Anaesthesia, Critical Care and Pain Medicine<br>51 Little France Crescent<br>Edinburgh, EH16 4SA<br>UNITED KINGDOM	West Midlands Research Ethics Commitee<br>West Midlands Research Ethics Commitee<br>Prospect House<br>Fishing Line Road<br>Enfield, Redditch B97 6EW<br>UNITED KINGDOM
1227	Dr. Mark Sair		Dr. Richard Cunningham<br>Dr. Michael Duffy<br>Dr. Peter MacNaughton	Intensive Care Unit, Level 4<br>Derriford Hospital<br>Plymouth, Devon PL6 8DH<br>UNITED KINGDOM	Cornwall & Plymouth REC<br>Room 101B, ITTC South Building<br>Tamar Science Park<br>Davy Road,<br>Derriford<br>Plymouth, PL6 8BX<br>UNITED KINGDOM<br><br>West Midlands Research Ethics Commitee<br>West Midlands Research Ethics Commitee<br>Prospect House<br>Fishing Line Road<br>Enfield, Redditch B97 6EW<br>UNITED KINGDOM

* Did not randomize subjects

090177e1816ca80c\Approved\Approved On: 06-Oct-2010 15:16

Center	Principal Investigator	Co-Investigator(s)	Sub-Investigator(s)	Address(es)	Institutional Review Board or Ethics Committee Address(es)

090177e1816ca80c\Approved\Approved On: 06-Oct-2010 15:16

* Did not randomize subjects

United States

Coordinating Investigators:
<None Entered>

Center	Principal Investigator	Co-Investigator(s)	Sub-Investigator(s)	Address(es)	Institutional Review Board or Ethics Committee Address(es)
1001	Dr. Addison Kemp May		Dr. Bryan Richard Collier<br>Dr. Ann Marie Conquest<br>Bryan A. Cotton<br>Dr. Jose Jesus Diaz Jr.<br>Dr. Oscar D. Guillamondegui<br>Dr. Oliver Lee Gunter Jr.<br>Dr. Jeffrey Scott Guy<br>Ms. Judith M. Jenkins<br>Dr. Gary T. Marshall<br>Dr. Vicente Alonso Mejia<br>Dr. Richard Stephen Miller<br>Dr. John Albert Morris Jr.<br>Dr. William P. Riordan	Hope Cambell<br>Pharmacy<br>Vanderbilt University Medical Center<br>1211 22nd Ave. South<br>Nashville, TN 37232<br>UNITED STATES<br><br>VANDERBILT UNIVERSITY MEDICAL CENTER<br>1161 21ST AVENUE SOUTH<br>NASHVILLE, TN 37232-7110<br>UNITED STATES	Vanderbilt University Institutional Review Board<br>Vanderbilt University Institutional Review Board<br>504 Oxford House<br>1313 21st Ave South<br>Nashville, TN 37232-4315<br>UNITED STATES
1010	Dr. Robert Jeffrey Kaner		Dr. John Paul Ayala<br>Steven Benkert<br>Dr. David A. Berlin<br>Dr. Joseph Thaddeus Cooke<br>Dr. Laura E. Crowley<br>Dr. Brian Gelbman<br>Dr. Ben-Gary Harvey<br>Dr. Rana Kaplan<br>Dr. Juhayna Kassem<br>Dr. Thomas K.C. King<br>Dr. Leah Lande<br>Francisco Pacheco<br>Dr. Abraham Sanders<br>Dr. Lynne M. Strasfeld<br>Dr. Meredith L. Turetz<br>Dr. Setu K. Vora<br>Dr. Dana Zappetti	New York Presbyterian Hosp/Weill Medical College of Cornell Univ<br>Ste 505<br>520 E 70th St<br>New York, NY 10021-9800<br>UNITED STATES	New York Presbyterian Hospital, Weill Medical College of Cornell University<br>Committee on Human Rights in Research<br>Suite DV-301<br>425 East 61st Street<br>New York, NY 10021<br>UNITED STATES

090177e1816ca80c\Approved\Approved On: 06-Oct-2010 15:16

090177e1816ca80c\Approved\Approved On: 06-Oct-2010 15:16

Center	Principal Investigator	Co-Investigator(s)	Sub-Investigator(s)	Address(es)	Institutional Review Board or Ethics Committee Address(es)
1005	Dr. Ali Albert El-Solh		Dr. Alan T. Aquilina<br>DR ARCHANA MISHRA<br>Dr. Lilibeth Pineda	Erie County Medical Center<br>462 Grider Street<br>Buffalo, NY 14215<br>UNITED STATES	University of Buffalo, The State University of New York<br>Universtiy of Buffalo Health Sciences Institutional Review Board<br>&<br>Institutional Animal Care and Use Committee<br>150 Parker Hall<br>University of Buffalo South Campus<br>Buffalo, NY 14214-8004<br>UNITED STATES
1003 *	Dr. Larry Marc Bush			JFK Medical Center<br>5301 South Congress Ave<br>Atlantis, FL 33462<br>UNITED STATES<br><br>South Florida Clinical Research<br>Suite #104<br>5503 South Congress Avenue<br>Atlantis, FL 33462<br>UNITED STATES	Western Institutional Review Board<br>3535 Seventh Avenue, SW<br>Olympia, WA 98502<br>UNITED STATES
1016 *	Dr. Devendra Natverlal Amin (Previous PI)<br>Dr. Ressa Marie McDonald		Dr. Devendra Natverlal Amin<br>Dr. Eli H. Freilich<br>Dr. John Alan Masson Jr.	Bay Area Chest Physicians<br>616 East St<br>Clearwater, FL 33756-3342<br>UNITED STATES<br><br>Morton Plant Hospital<br>300 Pinellas Street<br>Clearwater, FL 33756<br>UNITED STATES	Morton Plant Mease Healthcare Institutional Review Board<br>MS 143<br>207 Jeffords Street<br>Clearwater, FL 33756<br>UNITED STATES
1011 *	Dr. Richard Wayne Kearley		Dr. Ronald Ernest Fields	Our Lady of the Lake Medical Center<br>5000 Hennessy Blvd<br>Baton Rouge, LA 70808<br>UNITED STATES	Our Lady of the Lake Hospital, Inc Institutional Review Board<br>5000 Hennessy Blvd<br>Baton Rouge, LA 70808<br>UNITED STATES

* Did not randomize subjects

Center	Principal Investigator	Co-Investigator(s)	Sub-Investigator(s)	Address(es)	Institutional Review Board or Ethics Committee Address(es)
1015	Dr. Daniel George Lorch Jr.		Dr. Arthur Edward Graves<br>Dr. Thomas P. Hooker<br>Dr. Richard Shaw Powell	Brandon Regional Hospital<br>119 Oakfield Drive<br>Brandon, FL 33511<br>UNITED STATES<br><br>Pulmonary Associates of Brandon<br>Suite 102<br>910 Oakfield Drive<br>Brandon, FL 33511<br>UNITED STATES	Brandon Regional Hospital<br>Institutional Review Board<br>119 Oakfield Drive<br>Brandon, FL 33551<br>UNITED STATES
1004	Dr. Charles Mark Carpati		Dr. Mark E. Astriz<br>Michelle Glater<br>Dr. Linda A. Kirschenbaum	St. Vincent's Hospital - Manhattan<br>170 West 12th Street<br>New York, NY 10011<br>UNITED STATES	Biomedical Research Alliance of New York, LLC<br>Suite 100<br>225 Community Drive<br>Great Neck, NY 11021<br>UNITED STATES<br><br>Saint Vincent's Catholic Medical Centers<br>Integrated Scientific and Ethical Review Board<br>170 West 12th Street - Staff House<br>New York, NY 10011<br>UNITED STATES
1018	Dr. Gary J. Richmond		Vernon F. Appleby<br>Robert Kralejvich	Broward General Medical Center<br>1600 South Andrews Ave<br>Fort Lauderdale, FL 33316<br>UNITED STATES<br><br>Gary J Richmond MD PA<br>315 Southeast 14th Street<br>Fort Lauderdale, FL 33316<br>UNITED STATES	Broward Health Institutional Review Board<br>1600 South Andrews Avenue<br>Fort Lauderdale, FL 33316<br>UNITED STATES

090177e1816ca80c\Approved\Approved On: 06-Oct-2010 15:16

Center	Principal Investigator	Co-Investigator(s)	Sub-Investigator(s)	Address(es)	Institutional Review Board or Ethics Committee Address(es)
1023	Dr. William T. McGee		Dr. Lori E. Circeo<br>Dr. Thomas L. Higgins<br>Dr. Paul G. Jodka<br>Cynthia Kardos<br>Gerald Korona<br>Lori-Ann Kozikowski<br>Dr. Patrick T. Mailloux<br>Dr. Jay S. Steingrub<br>Dr. Gary J. Tereso<br>Mark A. Tidswell	Baystate Medical Center<br>759 Chestnut Ave<br>Springfield, MA 01199<br>UNITED STATES	Baystate Medical Center<br>Institutional Review Board<br>759 Chestnut Street<br>Springfield, MA 01199<br>UNITED STATES
1034	Dr. Keith M. Clance<br>(Previous PI)<br>Dr. Patrick Eugene Glen Wright Jr.		Rakesh Alva<br>Robert Byrum<br>Dr. Keith M. Clance<br>Daniel Feinstein<br>Jeremy Frens<br>Dr. Carmen Laura Gonzalez<br>Dr. Jeffery C. Hatcher<br>Murali Ramaswamy<br>Dr. David B. Simonds<br>Vineet Sood<br>Dr. Michael B. Wert<br>Dr. Wesam G. Yacoub	Moses Cone H. Memorial Hospital<br>1200 North Elm Street<br>Greensboro, NC 27401<br>UNITED STATES<br><br>Piedmont Respiratory Research Foundation<br>1200 North Elm Street<br>Greensboro, NC 27401<br>UNITED STATES	Moses Cone Health System<br>Office of Research Support, The Institutional Review Board (IRB)<br>1200 North Elm Street<br>Greensboro, NC 27401-1020<br>UNITED STATES
1021	Dr. Daniel Hugh Kett		Dr. Debra P. Fertel<br>Karen Marshall<br>Dr. Andrew Alan Quartin<br>Dr. Roland Milo Schein	University of Miami School of Medicine/Jackson Memorial Hospital<br>1611 NW 12th Avenue<br>Miami, FL 33136<br>UNITED STATES	University of Miami, School Of Medicine<br>Human Subjects Research Office<br>Tenth Floor, Suite 1000 (M-809)<br>1500 Northwest 12th Avenue<br>Miami, FL 33136<br>UNITED STATES

* Did not randomize subjects

090177e1816ca80c\Approved\Approved On: 06-Oct-2010 15:16

Center	Principal Investigator	Co-Investigator(s)	Sub-Investigator(s)	Address(es)	Institutional Review Board or Ethics Committee Address(es)
1020	Dr. Jose A. Vazquez<br>(Previous PI)<br>Dr. Patricia Diane Brown		Dr. John R. Ebright<br>Suzanne Woodrich	Detriot Receiving Hospital<br>4201 St. Antoine<br>Detroit, MI 48201<br>UNITED STATES<br><br>Harper University Hospital<br>3990 John R<br>5 Hudson<br>Detroit, MI 48210<br>UNITED STATES<br><br>Karmanos Cancer Center<br>4100 John R<br>Detroit, MI 48201<br>UNITED STATES<br><br>University Health Center<br>4201 St. Antoine<br>Detroit, MI 48201<br>UNITED STATES	Wayne State University Human Investigation Committee<br>101 East Alexandrine Building<br>Detroit, MI 48201<br>UNITED STATES
1026	Dr. James R. Dexter		Dr. Enrique G. Gil<br>Corrinne Jackson<br>Dr. Theodore L. Shankel	Redlands Community Hospital<br>350 Terracino Blvd<br>Redlands, CA 92373<br>UNITED STATES	Redlands Community Hospital<br>Ethics/IRB Committee<br>350 Terracina Boulevard<br>Redlands, CA 92373<br>UNITED STATES
1043	Dr. Scott H. Beegle		Dr. Rachel S. Hinerman<br>Dr. Jonathan M. Rosen<br>Dr. Thomas Clinton Smith<br>Dr. Simon Daniel Spivack	Albany Medical Center<br>47 New Scotland Ave<br>Albany, NY 12208<br>UNITED STATES	Western Institutional Review Board, Inc.<br>3535 Seventh Avenue, SW<br>Olympia, WA 98502<br>UNITED STATES

090177e1816ca80c\Approved\Approved On: 06-Oct-2010 15:16

Center	Principal Investigator	Co-Investigator(s)	Sub-Investigator(s)	Address(es)	Institutional Review Board or Ethics Committee Address(es)
1009	Dr. Christopher David Junker		Dr. Bruce Mathison Abell<br>Dr. Lakhmir S. Chawla<br>Dr. Guillermo Gutierrez<br>Dr. Michael Geren Seneff	George Washington University Hospital<br>900 23rd Street, NW<br>Washington, DC 20037<br>UNITED STATES	The George Washington University Committee on Human Research<br>Ross Hall Suite 612<br>2300 Eye Street Northwest<br>Washington, DC 20037<br>UNITED STATES
1017	Dr. Steven Douglas O'Marro		Dr. Sarah E. Boyd<br>Dr. Donald Richardson Graham<br>Holly E. Heffren<br>Dr. Douglas Ray Leigh<br>Praveen Mullangi<br>Robert W. Nelson<br>Jennifer Rogers<br>Dr. Mike Short<br>Amber S. Stanton	Memorial Medical Center<br>701 North First St.<br>Springfield, IL 62702<br>UNITED STATES<br><br>Springfield Clinic<br>1025 South Seventh Street<br>Springfield, IL 62703<br>UNITED STATES<br><br>Springfield Clinic Infectious Diseases<br>St. John's Pavilion<br>1st Floor<br>301 North Eighth Street<br>Springfield, IL 62701<br>UNITED STATES<br><br>Springfield Clinic Research Dept<br>Main Campus- West Building<br>Suite 4300<br>1025 S. 6th Street<br>Springfield, IL 62703<br>UNITED STATES<br><br>St. John's Hospital<br>800 East Carpenter St.<br>Springfield, IL 62702<br>UNITED STATES	Fox Commercial Institutional Review Board<br>Suite 202<br>133 South Fourth Street<br>Springfield, IL 62701<br>UNITED STATES

090177e1816ca80c\Approved\Approved On: 06-Oct-2010 15:16

090177e1816ca80c\Approved\Approved On: 06-Oct-2010 15:16

Center	Principal Investigator	Co-Investigator(s)	Sub-Investigator(s)	Address(es)	Institutional Review Board or Ethics Committee Address(es)
1022	Dr. Matthew Michael McCambridge<br>Stephen Charles Matchett (Previous PI)		Dr. Mark David Cipolle<br>Dr. Robert J. Kruklitis<br>Stephen Charles Matchett<br>Daniel E. Ray	Good Shepherd Specialty Hospital<br>LVH-M South Tower<br>2545 Shoenersville Road<br>Bethlehem, PA 18017<br>UNITED STATES<br><br>Lehigh Valley Hospital<br>1200 South Cedar Crest Blvd<br>Allentown, PA 18103<br>UNITED STATES	Lehigh Valley Hospital Institutional Review Board<br>Health Studies Research<br>6th Floor, Suite 38<br>17th and Crew Streets<br>Allentown, PA 18104<br>UNITED STATES
1032	Dr. William Paul Saliski Jr.		Dr. Fred D. Hunker<br>Dr. Geton D. Lorino<br>Dr. David R. Thrasher	Long Term Hospital of Montgomery<br>6th Floor, North Wing<br>1725 Pine Street<br>Montgomery, AL 36106<br>UNITED STATES<br><br>Montgomery Pulmonary Consultants, PA<br>1440 Narrow Lane Parkway<br>Montgomery, AL 36111<br>UNITED STATES	Baptist Health System Institutional Review Board<br>PO Box 244001<br>DeBoer Building Second Floor<br>301 Brown Springs Road<br>Montgomery, AL 36124-4001<br>UNITED STATES<br><br>Western Institutional Review Board, Inc.<br>3535 Seventh Avenue, SW<br>Olympia, WA 98502<br>UNITED STATES
1028 *	Dr. David Winslow Hines Jr.		Dr. Vishnu V. Chundi<br>Dr. Scott P. Neeley<br>Dr. Vijay Veerainder Yeldandi	Our Lady of Resurrection<br>5645 West Addison Street<br>Chicago, IL 60634<br>UNITED STATES	Our Lady of the Resurrection Medical Center Institutional Review Board<br>Our Lady of the Resurrection Medical Center<br>Institutional Review Board<br>5645 West Addison Street<br>Chicago, IL 60634<br>UNITED STATES

* Did not randomize subjects

Center	Principal Investigator	Co-Investigator(s)	Sub-Investigator(s)	Address(es)	Institutional Review Board or Ethics Committee Address(es)
1031	Dr. Ioana R. Preston		Dr. Carolyn M. D'Ambrosio<br>Dr. Nicholas Snowden Hill<br>Dr. Sean O'Reilly<br>Dr. Elaine Purcell<br>Samaan Rafeq<br>Joanne Rhofiry<br>Dr. Kari E. Roberts<br>Dr. Greg L. Schumaker<br>Dr. Archan Shah<br>Dr. Farhan Siddiqui	Pulmonayr Critical Care and Sleep Division<br>Tufts Medical Center<br>800 Washington Street # 257<br>Boston, MA 02111<br>UNITED STATES	Tufts Medical Center Institutional Review Board<br>Box 817<br>800 Washington Street<br>Boston, MA 02111<br>UNITED STATES
1033	Dr. Harold Clark Standiford		Dr. Mark Cowan<br>Dr. Graeme Neil Forrest	University of Maryland Medical System<br>22 South Greene Street<br>Baltimore, MD 21201<br>UNITED STATES<br><br>VA Maryland Health Care System<br>10 North Green Street<br>Baltimore, MD 21201<br>UNITED STATES	University of Maryland - Baltimore Institutional Reveiw Board<br>685 West Baltimore Street<br>Baltimore, MD 21201<br>UNITED STATES

090177e1816ca80c\Approved\Approved On: 06-Oct-2010 15:16

Center	Principal Investigator	Co-Investigator(s)	Sub-Investigator(s)	Address(es)	Institutional Review Board or Ethics Committee Address(es)
1036	Dr. Jeffrey Ross Hammersley		Dr. Amro Y. Al-Astal<br>Dr. Mohamad W. Al-Baghdadi<br>Dr. Ragheb Akram Assaly<br>John F. Blust<br>Andrew P. Fox<br>Dr. Dawn Alita Roberts Hernandez<br>Dr. Dan Emil Olson<br>Dr. Jamey J. Ruiz<br>Dr. Ahmad R. Saltagi<br>Dr. James Campbell Willey<br>Dr. Youngsook Yoon	University Medical Center<br>3000 Arlington Ave<br>Toledo, OH 43614<br>UNITED STATES	Medical University of Ohio at Toledo<br>Department for Human Research Protections/Institutional Review Board<br>Room 0106<br>3025 Arlington Avenue<br>CCE Building<br>Toledo, OH 43614-2470<br>UNITED STATES
1030 *	Dr. Boaz A. Markewitz		Dr. Srinivas B. Chakravarthy<br>Estelle Susan Harris<br>Dr. Robert Patrick	University of Utah Hospital<br>50 North Medical Drive<br>Salt Lake City, UT 84132<br>UNITED STATES	University of Utah IRB<br>512 RAB<br>75 South 2000 East<br>Salt Lake City, UT 84112<br>UNITED STATES

* Did not randomize subjects

090177e1816ca80c\Approved\Approved On: 06-Oct-2010 15:16

Center	Principal Investigator	Co-Investigator(s)	Sub-Investigator(s)	Address(es)	Institutional Review Board or Ethics Committee Address(es)
1038	Dr. William Rodriguez-Cintron		Edgardo J. Adono-Fontanez<br>Jose Adorno-Fernandez<br>Dr. Edwin A. Alicea-Colon<br>Dr. Miguel J. Boque-Santiago<br>Zulmari Campos-Santiago<br>Onix Cantres<br>Dr. Jesus R. Casal-Hidalgo<br>Dr. Jose L. Diaz-Pinazo<br>Dr. George P. Fahed-Inigo<br>Dr. Ricardo Fernandez-Gonzalez<br>Rosangela Fernandez-Medero<br>Dr. Carlos R. Garcia-Rodriguez<br>Dr. Glenda Gonzalez<br>Graciela Latalladi-Ortega<br>Dr. Marjery N. Lopez-Acevedo<br>Dr. Brenda L. Loubriel-Rivera<br>Biomaris Medina<br>Dr. Maria E. Ocasio-Tascon<br>Glorimar Santos-Llanos<br>Dr. Jose E. Torres-Palacios<br>Dr. Alfonso Torres-Palacios<br>Maria Del Mar Torres-Perez<br>Maria T. Vega-Martinez<br>Mark A. Vergara<br>Jason L. Willis	San Juan Veterans Affairs Medical Center<br>Pulmonary and Critical Care Medicine 111 East<br>10 Casia Street<br>San Juan, PR 00921-3201<br>UNITED STATES	Human Studies Subcommittee<br>San Juan Veterans Affairs Medical Center<br>Research and Development Committee<br>10 Casia Street<br>San Juan, PR 00921-3201<br>UNITED STATES

090177e1816ca80c\Approved\Approved On: 06-Oct-2010 15:16

Center	Principal Investigator	Co-Investigator(s)	Sub-Investigator(s)	Address(es)	Institutional Review Board or Ethics Committee Address(es)
1048	Dr. Melvin Lee Morganroth		Dr. William Minor Lile Bowerfind<br>Dr. Asha Narasimhan Chesnutt<br>Dr. Rhett James Cummings<br>Mary Alice Day<br>Christine Ebright<br>Dr. David Hotchkin<br>Dr. Marc Allan Jacobs<br>Michael L. Johnston<br>Dr. John Ford Keppel<br>Kim Lamorticella<br>Dr. Michael John Lefor<br>Dr. Louis Samuels Libby<br>Jane Manning<br>Dr. Richard John Maunder<br>Jackie Morganroth<br>Dr. James Randolph Patterson<br>Dr. Thomas Hyland Schaumberg<br>Dr. Michael David Skokan<br>Dr. Wayne M. Strauss<br>Dr. Karen Joan Wesenberg	Providence Portland Medical Center<br>4805 North East Glisan<br>Portland, OR 97213<br>UNITED STATES<br><br>The Oregon Clinic, P.C.<br>Suite 200<br>1111 NE 99th Avenue<br>Portland, OR 97220<br>UNITED STATES<br><br>Vibra Specialty Hospital<br>10300 NE Hancock Street<br>Portland, OR 97220<br>UNITED STATES	Providence Health System Institional Review Board<br>Building A Third Floor<br>5251 NE Glisan<br>Portland, OR 97213<br>UNITED STATES<br><br>Western Institutional Review Board, Inc.<br>3535 Seventh Ave SW<br>Olympia, WA 98508<br>UNITED STATES
1042	Dr. Domenick J. Reina		Dr. Margarita R. Cancio<br>Dr. Keith W. Chandler MD<br>Dr. Dorece G. Norris<br>Dr. Jose Eduardo Perez<br>Dr. Carlos J. Rozas MD<br>Dr. Mark Anthony Smith MD<br>Bonnie F. Tiemann<br>Dr. Scott S. Ubillos	St. Joseph's Hospital<br>3001 West Dr. Martin Luther King, Jr. Blvd<br>Tampa, FL 33607<br>UNITED STATES	St. Joseph's Hospital Institutional Review Board<br>3001 West Dr. Martin Luther King, Jr. Boulevard<br>Tampa, FL 33607<br>UNITED STATES

090177e1816ca80c\Approved\Approved On: 06-Oct-2010 15:16

Center	Principal Investigator	Co-Investigator(s)	Sub-Investigator(s)	Address(es)	Institutional Review Board or Ethics Committee Address(es)
1050 *	Dr. Cordelia V. Sharma		Dr. Arnold W. Berlin<br>Dr. Randall P. Owen<br>Dr. Thanjavur S. Ravikumar	Montifore Medical Center<br>111 East 210th Street<br>Bronx, NY 10467<br>UNITED STATES	Montefiore Medical Center<br>Office of Research and Sponsored Programs<br>111 East 210th Street<br>Bronx, NY 10467<br>UNITED STATES
1051	Dr. Michael Steven Sherman		Karim Djekidel<br>anas hadeh<br>Jeffrey Hoag<br>Denise Lai<br>Dr. Herbert Patrick<br>Dr. Siva Kumar Ramachandran<br>Dr. Edward S. Schulman	Hahnemann University Hospital<br>Broad and Vine Streets<br>Philadelphia, PA 19102<br>UNITED STATES	Western Institutional Review Board, Inc.<br>3535 Seventh Avenue SW<br>Olympia, WA 98502-5010<br>UNITED STATES
1039 *	Dr. Priscilla Bayani Sioson		Dr. Julie T. Antique<br>Dr. Debra Lee Rainey	Jackson-Madison County General Hospital<br>708 West Forest Avenue<br>Jackson, TN 38301<br>UNITED STATES<br><br>West Tennessee Transitional Care<br>670 Skyline Drive<br>Jackson, TN 38301<br>UNITED STATES	Jackson-Madison County General Hospital IRB<br>708 West Forest Avenue<br>Jackson, TN 38301<br>UNITED STATES

* Did not randomize subjects

090177e1816ca80c\Approved\Approved On: 06-Oct-2010 15:16

Center	Principal Investigator	Co-Investigator(s)	Sub-Investigator(s)	Address(es)	Institutional Review Board or Ethics Committee Address(es)
1040	Daniel J. Naughton<br>Dr. Aaron Michael Scifres (Previous PI)<br>Dr. Carl A. Freeman (Previous PI)<br>Dr. Lonnie Warren Frei (Previous PI)		Dr. Jeffrey Bailey<br>Craig Dedert<br>Mr. John A. Evans<br>Dr. Carl A. Freeman<br>Brent Ibata<br>Shannon G. Lehner<br>Kathryn K. Lindsay<br>Kevin Mahoney<br>Dr. Sapoora Manshaii<br>Dr. George Marion Matuschak<br>Dr. Nahel N. Saied<br>Anna Schmidt<br>Dr. Aaron Michael Scifres<br>Jane E. Tenquist<br>Dr. Catherine Marie Wittgen<br>Dr. Hui Yuan	Saint Louis University<br>3635 Vista Avenue at Grand Boulevard<br>St. Loius, MO 63110<br>UNITED STATES	Saint Louis University Hospital<br>Biomedical Institutional Review Board<br>3556 Caroline Street<br>Room 110<br>St. Louis, MO 63104<br>UNITED STATES
1046	Dr. Robert Curtis Hyzy		Dr. Robert H. Bartlett<br>Dr. Ronald E. Dechert<br>Dr. Christopher L. Fraley<br>Dr. Steven Eldon Gay<br>Dr. Andrew L. Rosenberg	University of Michigan Health System<br>Adult Hospital<br>1500 East Medical Center Drive<br>Ann Arbor, MI 48109-0331<br>UNITED STATES	University of Michigan Medical School<br>Institutional Review Board for Human Subjects Research<br>4558 Kresge Medical Research Building One<br>200 Zina Pitcher<br>Ann Arbor, MI 48109<br>UNITED STATES

090177e1816ca80c\Approved\Approved On: 06-Oct-2010 15:16

Center	Principal Investigator	Co-Investigator(s)	Sub-Investigator(s)	Address(es)	Institutional Review Board or Ethics Committee Address(es)
1047	Dr. John Edward Mazuski		Dr. Timothy George Buchman<br>Dr. Craig M. Coopersmith<br>Steven E. Finkelstein<br>Dr. Eric Jacobsohn<br>Dr. Douglas Schuerer	The Barnes-Jewish Hospital<br>One Barnes Jewish Hospital Plaza<br>St. Louis, MO 63110<br>UNITED STATES<br><br>Washington University School of Medicine<br>Campus Box 8109<br>660 South Euclid Avenue<br>Saint Louis, MO 63110-1010<br>UNITED STATES	Washington University Medical Center IRB<br>Washington University Medical Center<br>Human Studies Committee<br>660 South Euclid Avenue<br>Box 8089<br>St. Louis, MO 63110<br>UNITED STATES
1052	Dr. William Thomas Dickey Jr.		Dr. David Matthew Cantrell<br>Dr. Michael Patrick Witt	BAYLOR MEDICAL CENTER AT IRVING CLINICAL RESEARCH<br>1901 NORTH MACARTHUR BOULEVARD<br>IRVING, TX 75061<br>UNITED STATES	Baylor Research Institute<br>Institutional Review Board<br>Suite 125<br>3434 Live Oak<br>Dallas, TX 75204<br>UNITED STATES
1007	Dr. Bruce C. Friedman		Dr. S M Abu Zaheed Hassan<br>Dr. Robert F. Mullins<br>Dr. Joseph R. Shaver<br>Dr. Amy McElroy Sprague	Doctors Hospital<br>3651 Wheeler Road<br>Augusta, GA 30909<br>UNITED STATES<br><br>Joseph M. Still Research Foundation, Inc.<br>4A George C. Wilson Court<br>Augusta, GA 30909<br>UNITED STATES	Doctors Hospital Augusta<br>Institutional Review Board<br>3651 Wheeler Road<br>Augusta, GA 30909<br>UNITED STATES

090177e1816ca80c\Approved\Approved On: 06-Oct-2010 15:16

Center	Principal Investigator	Co-Investigator(s)	Sub-Investigator(s)	Address(es)	Institutional Review Board or Ethics Committee Address(es)
1008	Dr. Azmi Draw (Previous PI)<br>Dr. Raul Nakamatsu<br>Dr. Subin Jain (Previous PI)		Mary Beth Allen<br>Dr. Marty Allen<br>Amirali Ali Amjadi<br>Dr. Forest W. Arnold<br>Dr. Azmi Draw<br>Uchenna Ezike<br>Dr. Juan J. Guardiola<br>Rama Kapoor<br>Dr. Pauline A. Lett<br>Dr. Maricar F. Malinis<br>Paula Peyrani<br>Dr. Julio A. Ramirez<br>Denise Rueff<br>Ginny Sciortino<br>Inemesit Umoren	University of Louisville<br>Division of Infectious Deseases<br>Suite 380<br>501 East Broadway<br>Louisville, KY 40202<br>UNITED STATES<br><br>Veteran Affairs Medical Center<br>800 Zorn Avenue<br>Louisville, KY 40206<br>UNITED STATES	Veterens Affairs Medical Center<br>Human Studies Subcomittee<br>Suite 151<br>800 Zorn Avenue<br>Louisville, KY 40206<br>UNITED STATES

090177e1816ca80c\Approved\Approved On: 06-Oct-2010 15:16

090177e1816ca80c\Approved\Approved On: 06-Oct-2010 15:16

Center	Principal Investigator	Co-Investigator(s)	Sub-Investigator(s)	Address(es)	Institutional Review Board or Ethics Committee Address(es)
1012	Dr. Paul Anthony Kearney Jr.		Ms. Marietta Barton-Baxter<br>Stephanie Baugh<br>Dr. Rolando R. Berger<br>Dr. Andrew Bernard<br>Anthony Bottiggi<br>Dr. Bernard Boulanger<br>Dr. Phillip K. Chang<br>Dr. Aaron M. Cook<br>Dr. Jeffrey P. Coughenour<br>Nannette Freeman<br>Rebecca Geyer<br>Tracy Jackson<br>Brittany Kaiser<br>Joy Kimbrough<br>Christie Merrill<br>Lauren Morrow<br>Levi D. Procter<br>Julia Reed<br>Ms. Anna K. Rockich<br>Danielle Sager<br>Rebecca Shelton<br>Matthew E. Simpson<br>Pam Stafford<br>Lauren Wiggington-White<br>P. Shane Winstead<br>Marta Wood<br>Bryan Yankey	University of Kentucky<br>800 Rose Street<br>Lexington, KY 40536<br>UNITED STATES<br><br>University of Kentucky Chandler Medical Center<br>800 Rose Street<br>Lexington, KY 40536<br>UNITED STATES	University of Kentucky Hospital<br>University of Kentucky Hospital/Medical Institutional Review Board/Office of Research Integrity<br>315 Kinkead Hall<br>Lexington, KY 40506-0057<br>UNITED STATES
1013	Dr. William Kien-ki Lau		Dr. Stuart Sugihara<br>Renee Tichy	Kuakini Medical Center<br>347 North Kuakini Street<br>Honolulu, HI 96817<br>UNITED STATES	Kuakini Research and Institutional Review Committee<br>347 North Kuakini Street<br>Honolulu, HI 96817<br>UNITED STATES

* Did not randomize subjects

Center	Principal Investigator	Co-Investigator(s)	Sub-Investigator(s)	Address(es)	Institutional Review Board or Ethics Committee Address(es)
1045	Dr. Joseph Harrison Patton Jr.		Dr. Craig F Copeland<br>Dr. Noreen Durrani<br>Dr. Erin O. Field<br>Dr. Harriette Mathilda Horst<br>Dr. Mike McCann<br>Dr. Usman Waheed	Henry Ford Health System<br>2799 West Grand Boulevard<br>Detroit, MI 48202<br>UNITED STATES	Henry Ford Health System,<br>Institutional Review Board<br>CFP-Basement 046<br>2799 West Grand Boulevard<br>Detroit, MI 48202<br>UNITED STATES
1044	Dr. Joann Bennett		Dr. Aristides P. Assimacopoulos<br>Susan E. Baumgart-Weaver<br>Dr. Rizan A. Hajal<br>Dr. Brian T. Hurley<br>Julie M. Warren-Kittlesrud	Avera Downtown Center<br>Suite 117<br>300 North Dakota Avenue<br>Sioux Falls, SD 57104<br>UNITED STATES<br><br>Avera McKennan Hospital<br>Box 5045<br>800 East 21st Street<br>Sioux Falls, SD 57117-5045<br>UNITED STATES<br><br>Avera Research Institute<br>2020 South Norton Avenue<br>Sioux Falls, SD 57105<br>UNITED STATES<br><br>Pulmonary Assoc./Avera Health<br>Suite 500<br>911 East 20th Street<br>Sioux Falls, SD 57105<br>UNITED STATES	Avera Institutional Review Board<br>3900 Avera Drive<br>Sioux Falls, SD 57108<br>UNITED STATES
1068 *	Dr. Geetika Nisha Sood		Dr. Bartholomew Raymond Bono<br>Dr. Jorge Ignacio Mora<br>Dr. Jerry Marc Zuckerman	ALBERT EINSTEIN MEDICAL CENTER<br>5501 OLD YORK ROAD<br>PHILADELPHIA, PA 19141<br>UNITED STATES	Albert Einstein Healthcare Network<br>Institutional Review Board<br>Korman Building, Room 100<br>5501 Old York Road<br>Philadelphia, PA 19141<br>UNITED STATES

* Did not randomize subjects

090177e1816ca80c\Approved\Approved On: 06-Oct-2010 15:16

Center	Principal Investigator	Co-Investigator(s)	Sub-Investigator(s)	Address(es)	Institutional Review Board or Ethics Committee Address(es)
1070	Dr. Jorge I. Cue<br>Dr. Robert George Martindale (Previous PI)		Dr. Gina L. Adrales<br>Mary M. Bannan<br>Dr. Colville H. B. Ferdinand<br>Dr. Thomas R. Gadacz<br>Dr. Michael L. Hawkins<br>Dr. David M. Killough<br>Dr. Bruce Vischer Macfadyen Jr.<br>Dr. John D. Mellinger<br>Mary Anne Park<br>Brenda B. Rosson<br>Carol A. Smith<br>Dayna Swinson	Medical College of Georgia<br>1120 15th Street<br>Augusta, GA 30912<br>UNITED STATES	Human Assurance Committee<br>Medical College of Georgia<br>CJ-2103<br>1120 15th Street<br>Augusta, GA 30912<br>UNITED STATES
1072 *	Dr. Robert Michael Aris		Dr. Shannon Stewart Carson<br>Dr. Raymond D. Coakley<br>Heather P. Krumnacher<br>Dr. Isabel P. Neuringer	University of North Carolina Hospitals<br>101 Manning Drive<br>Chapel Hill, NC 27514<br>UNITED STATES	The University of North Carolina at Chapel Hill<br>Office of Human Research Ethics<br>Biomedical Institutional Review Board<br>Medical School Building 52<br>Campus Box 7097<br>Chapel Hill, NC 27599-7097<br>UNITED STATES

* Did not randomize subjects

090177e1816ca80c\Approved\Approved On: 06-Oct-2010 15:16

090177e1816ca80c\Approved\Approved On: 06-Oct-2010 15:16

Center	Principal Investigator	Co-Investigator(s)	Sub-Investigator(s)	Address(es)	Institutional Review Board or Ethics Committee Address(es)
1073	Rod L. Elliott-Mullens		Dr. Guido J. Calderon	Central Texas Medical Center<br>1301 Wonder World Drive<br>San Marcos, TX 78666<br>UNITED STATES<br><br>Central Texas Pulmonary<br>Suite 308<br>1305 Wonder World Drive<br>San Marcos, TX 78666<br>UNITED STATES<br><br>TEAM Research of Sequin<br>1117 East Court Street<br>Sequin, TX 78155<br>UNITED STATES	Western Institutional Review Board, Inc.<br>3535 7th Avenue, Southwest<br>Olympia, WA 98502-5010<br>UNITED STATES
1075	Dr. David C. Kaufman		Dr. Michael J. Apostolakos<br>Stephen A. Bean<br>Dr. Joseph W. Dooley<br>Dr. Carolyn E. Jones	University of Rochester Medical Center/Strong Memorial Hospital<br>601 Elmwood Avenue<br>Rochester, NY 14642<br>UNITED STATES	Western Institutional Review Board, Inc.<br>3535 Seventh Avenue, SW<br>Olympia, WA 98502<br>UNITED STATES
1078 *	Dr. Miren A. Schinco-Schaffer		Dr. Margaret Mary Griffen<br>Dr. Andrew Kerwin	Faculty Clinic<br>653 West 8th Street<br>Jackson, FL 32209<br>UNITED STATES<br><br>University of Florida Health Science Center-Jacksonville<br>SHANDS Jacksonville Medical Center<br>655 West 8th St.<br>Jacksonville, FL 32209<br>UNITED STATES	Western Institutional Review Board, Inc.<br>3535 Seventh Avenue, SW<br>Olympia, WA 98502<br>UNITED STATES

* Did not randomize subjects

Center	Principal Investigator	Co-Investigator(s)	Sub-Investigator(s)	Address(es)	Institutional Review Board or Ethics Committee Address(es)
1084 *	Dr. Paul Joseph Scheinberg		Dr. Juan C. Armstrong<br>Dr. Kathryn M. McMinn	Atlanta Pulmonary Group, LLC<br>Suite 390<br>5667 Peachtree Dunwoody Rd.<br>Atlanta, GA 30342<br>UNITED STATES<br><br>St. Joseph's Hospital of Atlanta<br>5665 Peachtree Dunwoody Road<br>Atlanta, GA 30342<br>UNITED STATES	Saint Joseph's Hospital of Atlanta<br>Instutitional Review Board<br>Suite 685<br>5673 Peachtree Dunwoody Road<br>Atlanta, GA 30342<br>UNITED STATES
1085 *	Dr. Paul Peniston Cook		Dr. Titu Das<br>Dr. Carlos Guerra<br>Dr. Thomas Michael Kerkering<br>Dr. Ricardo A. Maldonado<br>Madhvi Rana<br>Dr. Kashif Raza<br>Dr. Dawd Said Siraj<br>Tammy Worden	Brody School of Medicine at East Carlolina University<br>Doctor's Park 6A<br>Greenville, NC 27834<br>UNITED STATES<br><br>Brody School of Medicine at East Carolina University (ECU)<br>600 Moye Boulevard<br>Greenville, NC 27834<br>UNITED STATES<br><br>Pitt County Memorial Hospital<br>2100 Stantonsburg Road<br>Greenville, NC 27834<br>UNITED STATES	University and Medical Center IRB<br>East Carolina University<br>LSB 104<br>Ed Warren Life Sciences Building<br>600 Moye Boulevard<br>Greenville, NC 27834<br>UNITED STATES

090177e1816ca80c\Approved\Approved On: 06-Oct-2010 15:16

Center	Principal Investigator	Co-Investigator(s)	Sub-Investigator(s)	Address(es)	Institutional Review Board or Ethics Committee Address(es)
1087	Dr. Graciella Soto (Previous PI)<br>Dr. Hidenobu Shigemitsu<br>Dr. Tony N. Hodges (Previous PI)		Dr. Adupa P. Rao	LAC/USC Medical Center<br>1200 North State Street<br>Los Angeles, CA 90033<br>UNITED STATES<br><br>USC University Hospital, USC Cardiothoracic Transplant Program<br>Suite 4300<br>1520 San Pablo Street<br>Los Angeles, CA 90033<br>UNITED STATES	Institutional Review Board<br>LAC/USC Medical Center<br>Intern's residence Dorm #425<br>2020 Zonal Avenue<br>Los Angelas, CA 90033<br>UNITED STATES
1089	Dr. Walid Fayez Khayr			Veterans Affairs Medical Center North Chicago<br>3001 Green Bay Road<br>North Chicago, IL 60064<br>UNITED STATES	Human Studies Subcommittee<br>5th Avenue and Roosevelt Road<br>Hines, IL 60141<br>UNITED STATES
1090 *	Dr. Robert Lee Swords			St. John's Clinic-Infectious Disease Associates<br>Suite 2955<br>1900 South National Avenue<br>Springfield, MO 65804<br>UNITED STATES<br><br>St. John's Medical Research<br>3231 South National Avenue<br>Springfield, MO 65807<br>UNITED STATES<br><br>St. John's Regional Health Center<br>1235 East Cherokee Street<br>Springfield, MO 65804<br>UNITED STATES	St. John's Institutional Review Board<br>1235 East Cherokee Street<br>Springfield, MO 65804<br>UNITED STATES

* Did not randomize subjects

090177e1816ca80c\Approved\Approved On: 06-Oct-2010 15:16

090177e1816ca80c\Approved\Approved On: 06-Oct-2010 15:16

Center	Principal Investigator	Co-Investigator(s)	Sub-Investigator(s)	Address(es)	Institutional Review Board or Ethics Committee Address(es)
1134	Dr. Jerome F. Levine		Dr. Bindu Balani<br>Dr. Steven A. Blau<br>Dr. Christina Emanuela Cicogna<br>Samit Desai<br>Dr. Peter Alan Gross<br>Ossama I. Ikladios<br>Dr. Emanuele Andrew Santomauro<br>Dr. Rani Sebti<br>Dr. Steven Jay Sperber	Center for Infectious Diseases<br>Hackensack University Medical Center<br>Suite 507<br>20 Prospect Avenue<br>Hackensack, NJ 07601<br>UNITED STATES<br><br>Hackensack University Medical Center<br>30 Prospect Avenue<br>Hackensack, NJ 07601<br>UNITED STATES	Western Institutional Review Board, Inc.<br>WIRB<br>3535 Seventh Avenue, Southwest<br>Olympia, WA 98502-5010<br>UNITED STATES
1137 *	Dr. Kevin N. Foster		Dr. Tammy R. Kopelman	MARICOPA MEDICAL CENTER<br>2601 EAST ROOSEVELT<br>PHOENIX, AZ 85008<br>UNITED STATES	Maricopa Integrated Health System Institutional Review Board<br>2601 East Roosevelt<br>Phoenix, AZ 85008<br>UNITED STATES
1138 *	Dr. Dennis Eugene Amundson (Previous PI)<br>Dr. John Scott Parrish		Dr. Mary F. Bavaro<br>Dr. Matthew E. Boland	Naval Medical Center San Diego<br>34800 Bob Wilson Drive<br>San Diego, CA 92134<br>UNITED STATES	Naval Medical Center San Diego<br>Suite 5<br>34800 Bob Wilson Drive<br>San Diego, CA 92134-5000<br>UNITED STATES
1144	Dr. Paul E. Bankey		Dr. Julius D. Cheng	University of Rochester Medical Center Strong Memorial Hospital<br>601 Elmwood Ave<br>Rochester, NY 14642-8410<br>UNITED STATES	Western Institutional Review Board, Inc.<br>3535 Seventh Avenue, SW<br>Olympia, WA 98502<br>UNITED STATES

* Did not randomize subjects

090177e1816ca80c\Approved\Approved On: 06-Oct-2010 15:16

Center	Principal Investigator	Co-Investigator(s)	Sub-Investigator(s)	Address(es)	Institutional Review Board or Ethics Committee Address(es)
1145	Dr. John Guy Mastronarde		Imad Joseph Bout-Akl<br>Scott Kenneth Aberegg<br>Dr. Naeem A. Ali<br>Dr. James Norman Allen Jr.<br>Beth Besecker<br>Dr. Nitin Yogendra Bhatt<br>Emily Jane Brawner<br>Benjamin Bringardner<br>Aaron Scott Bruns<br>Dr. Elliott David Crouser<br>Kiran Kumar Devulapally<br>Dr. Philip Tomas Diaz<br>Leroy Essig<br>Matthew Exline<br>Michael Ezzie<br>Robert P. Fudge<br>Michael B Green<br>Bradley R Harrold<br>Dr. Stephen Hoffmann<br>Daniel Vassilev Iltchev<br>Steven Kadiev<br>Rami Khayat<br>Stephen E. Kirkby<br>Dr. Maria R. Lucarelli<br>David R. Nunley<br>James M O'Brien<br>Jonathan Parsons<br>Troy A Schaffernocker<br>Dr. Leanne Kay Strack<br>Martin A Valdivia-Arenas<br>Karen L. Wood	The Ohio State University Medical Center<br>410 West Tenth Avenue<br>Columbus, OH 43210<br>UNITED STATES	Western Institutional Review Board<br>3535 Seventh Avenue, SW<br>Olympia, WA 98502-5010<br>UNITED STATES
1146 *	Dr. Steven D. Tennenberg		Dr. Walter A. Salwen	John D. Dingell VA Medical Center<br>Department of Surgery (11S)<br>4646 John R<br>Detroit, MI 48201<br>UNITED STATES	Wayne State University<br>Human Investigation Committtee<br>University Health Center, 6G<br>4201 St. Antoine Blvd<br>Detroit, MI 48201<br>UNITED STATES

* Did not randomize subjects

090177e1816ca80c\Approved\Approved On: 06-Oct-2010 15:16

Center	Principal Investigator	Co-Investigator(s)	Sub-Investigator(s)	Address(es)	Institutional Review Board or Ethics Committee Address(es)
1147	Dr. Joseph M. Gastaldo		Dr. Ian McNicoll Baird<br>Dr. Simrit K. Bhullar<br>Joshua E. Bitter<br>Dr. Edward Michael Cordasco Jr.<br>Barbara Danenbergs<br>Caleb Mackey<br>Brian R. Zeno	Remington-Davis, Inc.<br>Suite 106A<br>1335 Dublin Road<br>Columbus, OH 43215<br>UNITED STATES<br><br>Riverside Infections Consultants<br>Suite 3020<br>3555 Olentangy River Road<br>Columbus, OH 43214<br>UNITED STATES<br><br>Riverside Methodist Hospital<br>3535 Olentangy River Road<br>Columbus, OH 43214<br>UNITED STATES<br><br>Riverside Pulmonary Associates, Inc.<br>Suite 201<br>3535 Olentangy River Road<br>Columbus, OH 43214<br>UNITED STATES	Riverside Methodist Hospital<br>Grady Memorial Hospital,<br>Institutional Review Board<br>Suite 404<br>3545 Olentangy River Road<br>Columbus, OH 43214<br>UNITED STATES
1148	Dr. Stephen E. Olvey		Dr. Enrique Ginzburg	University of Miami School of Medicine/Jackson Memorial Hospital<br>1601 NW 12th Avenue<br>Miami, FL 33136<br>UNITED STATES	University of Miami, School Of Medicine<br>Human Subjects Research Office<br>Tenth Floor, Suite 1000 (M-809)<br>1500 Northwest 12th Avenue<br>Miami, FL 33136<br>UNITED STATES

* Did not randomize subjects

090177e1816ca80c\Approved\Approved On: 06-Oct-2010 15:16

Center	Principal Investigator	Co-Investigator(s)	Sub-Investigator(s)	Address(es)	Institutional Review Board or Ethics Committee Address(es)
1156	Dr. Marianne Eva Cinat		Dr. Cristobal Barrios<br>Mr. Carlos Chavez<br>Dr. Matthew O. Dolich<br>Lorene Kong<br>Dr. Christopher T. Lane<br>Dr. Michael Edward Lekawa<br>Dr. Chirag Vipin Patel	University of California Irvine Medical Center<br>101 The City Drive<br>Orange, CA 92868<br>UNITED STATES	University of California, Irvine<br>Office of Research Administration<br>Suite 300<br>4199 Campus Drive<br>Irvine, CA 92697-7600<br>UNITED STATES
1157	Dr. Liziamma George		Dr. Teena Abraham<br>Dr. Ayman Asaad Bishay<br>Dr. Frew H. Gebreab	New York Methodist Hospital<br>506 Sixth Street<br>Brooklyn, NY 11215<br>UNITED STATES	New York Methodist Hospital<br>Institutional Reveiw Committee<br>506 Sixth Street<br>Brooklyn, NY 11215<br>UNITED STATES
1160 *	Dr. Larry I. Emdur		Dr. Gang Bao<br>Dr. Parvathi Tiruviluamala	Alvarado Hospital Medical Center<br>6655 Alvarado Rd.<br>San Diego, CA 92120<br>UNITED STATES	Alvarado Hospital Institutional Review Board<br>6655 Alvarado Road<br>San Diego, CA 92120<br>UNITED STATES
1162	Dr. Ronald L. Ciubotaru		Dr. Darryl L. Adler<br>Nur-E-Ain Mirza<br>Anita Mimi Nacario<br>Mervyn Richardson<br>Dr. Mihal Smina<br>Dr. Richard L. Stumacher	St. Barnabas Hospital<br>4422 3rd Ave<br>Bronx, NY 10457<br>UNITED STATES	St. Barnabas Hospital<br>Institutional Review Board<br>Mills Building, Fourth Floor<br>4422 Third Avenue<br>Bronx, NY 10457-2594<br>UNITED STATES
1163 *	Dr. David L. Balfe		Dr. Michael Ian Lewis<br>Dr. Aruna Rekha Murthy<br>Dr. Vivian Shirvani	Cedars-Sinai Medical Center #6732<br>8700 Beverly Boulevard<br>Los Angeles, CA 90048<br>UNITED STATES	Cedars Sinai Medical Center<br>Institutional Review Board<br>8700 Beverly Blvd.<br>North Towe Plaza, 2015<br>Los Angeles, CA 90048<br>UNITED STATES

* Did not randomize subjects

Center	Principal Investigator	Co-Investigator(s)	Sub-Investigator(s)	Address(es)	Institutional Review Board or Ethics Committee Address(es)
1165 *	Dr. Arthur P. Wheeler		Dr. Gordon Raphael Bernard<br>Teresa M. Welch	Drug Shipment Address/Vanderbilt University Medical Center<br>Investigational Drug Service<br>Attn: Lori M. Choate<br>1161 21st Avenue South, VUH Room B-101<br>Nashville, TN 37232<br>UNITED STATES<br><br>Vanderbilt University Medical Center<br>1161 21st Avenue South<br>Nashville, TN 37232<br>UNITED STATES	VANDERBILT UNIVERSITY MEDICAL CENTER<br>Vanderbilt University Institutional Review Board<br>D-3232 Medical Center North<br>Nashville, TN 37232<br>UNITED STATES
1168	Dr. Peter Kenneth Linden (Previous PI)<br>Dr. Sachin Yende		Karl Crevar<br>Joseph M. Darby<br>Dr. Scott R. Gunn<br>Dr. Jason E. Moore<br>Juan B. Ochoa<br>Dr. Ramesh Vankataraman	UPMC-Presbyterian<br>200 Lothrop Street<br>Pittsburgh, PA 15213<br>UNITED STATES	University of Pittsburgh<br>Institutional Review Board<br>Ground Level<br>3500 Fifth Avenue<br>Pittsburgh, PA 15213<br>UNITED STATES
1171	Dr. Benjamin Dave Margolis		Dr. Juan R. Herena<br>Dr. Anthony M. Marinelli Jr.	West Suburban Hospital Medical Center<br>Ste 3000<br>1 Erie Court<br>Oak Park, IL 60302-2566<br>UNITED STATES	Western Institutional Review Board<br>3535 Seventh Avenue, SW<br>Olympia, WA 98502<br>UNITED STATES
1172	Dr. Luis J. Lugo-Velez		Dr. Omar L. Caban-Acevedo<br>Dr. Domingo Chardon-Feliciano<br>Dr. Federico Montealegre	Ponce School of Medicine<br>388 Zona Industila Reparada 2<br>Ponce, PR 00716<br>UNITED STATES	Western Institutional Review Board<br>3535 Seventh Avenue, SW<br>Olympia, WA 98502-5010<br>UNITED STATES

* Did not randomize subjects

090177e1816ca80c\Approved\Approved On: 06-Oct-2010 15:16

Center	Principal Investigator	Co-Investigator(s)	Sub-Investigator(s)	Address(es)	Institutional Review Board or Ethics Committee Address(es)
1176	Dr. John Richard Siever		Dr. Gregory Scott Ahearn<br>Carolyn Beno<br>Dr. Jeremy Feldman<br>Dr. James R. Forseth<br>Dr. Muhammad Raza<br>Dr. Richard Sue<br>Dr. Shawn E. Wright	St. Joseph's Hospital and Medical Center<br>SELECT Specialty Hospital<br>350 West Thomas Road<br>Phoenix, AZ 85013<br>UNITED STATES	St. Joseph's Hospital and Medical Center, Institutional Review Board<br>350 West Thomas Road<br>Phoenix, AZ 85013<br>UNITED STATES<br><br>Western Institutional Review Board<br>3535 Seventh Avenue, SW<br>Olympia, WA 98502<br>UNITED STATES
1177	Dr. Suresh K. Agarwal Jr.		John Abbensetts<br>Dawn M. Angelini<br>Dr. Peter A. Burke<br>Dr. Philip A. Cohen<br>Nancy Connors<br>Dr. Richard C. Dennis<br>Dr. Timothy A. Emhoff<br>Dr. Andrew I. Glantz<br>Patricia Harrison<br>Dr. Erwin F. Hirsch<br>Lauren Howard<br>Dr. Ishaq I. Lat<br>Eric Mahoney<br>Janet Orf<br>Amanda Young	Boston University Medical Center<br>One Boston Medical Center Place<br>Dowling 2 South<br>Boston, MA 02118<br>UNITED STATES	Western Institutional Review Board<br>3535 Seventh Avenue, SW<br>Olympia, WA 98502-5010<br>UNITED STATES

090177e1816ca80c\Approved\Approved On: 06-Oct-2010 15:16

Center	Principal Investigator	Co-Investigator(s)	Sub-Investigator(s)	Address(es)	Institutional Review Board or Ethics Committee Address(es)
1179	Dr. Richard W. Snyder		Mr. Chad Barefoot<br>Mary Beredjiklian<br>Dr. Christopher G. Bosse<br>Dr. G. Chris Christensen<br>Dr. Joseph G. Crocetti<br>Dr. Richard E. Friedenheim<br>Mary H. Kelly<br>Dr. Henry S. Mishel<br>Ms. Barbara Pugh<br>Dr. Conrad C. Reed<br>Dr. Bruce Roy<br>Dr. Stanley P. Silverman<br>Dr. William Louis Ward	Abington Memorial Hospital<br>1200 Old York Road<br>Abington, PA 19001<br>UNITED STATES	Abington Memorial Hospital<br>Institutional Review Board<br>1200 Old York Road<br>Abington, PA 19001-3788<br>UNITED STATES
1181	Julin F. Tang		Mark S. Siobal<br>Dr. Lisa Gail Winston	San Francisco General Hospital<br>1001 Protrero Avenue<br>San Francisco, CA 94110<br>UNITED STATES	University of California, San Francisco<br>Office of Research, Committee on Human Research<br>Suite 315<br>3333 California Avenue<br>San Francisco, CA 94143-0962<br>UNITED STATES
1182	Dr. Stephen Leonard Barnes		Mr. Richard D. Branson<br>Dr. Kenneth R. Davis Jr.<br>Dr. Jay Albert Johannigman<br>Dr. Tim Pritts<br>Dr. Betty Tsuei	University Hospital, Inc.<br>234 Goodman Street<br>Cincinnati, OH 45219<br>UNITED STATES<br><br>University of Cincinnati Medical Center/Department of Surgery<br>ML0558<br>231 Albert Sabin Way<br>Cincinnati, OH 45267-0558<br>UNITED STATES	University of Cincinnati Medical Center Institutional Review Board<br>G08 Wherry Hall<br>Cincinnati, OH 45267<br>UNITED STATES

* Did not randomize subjects

090177e1816ca80c\Approved\Approved On: 06-Oct-2010 15:16

Center	Principal Investigator	Co-Investigator(s)	Sub-Investigator(s)	Address(es)	Institutional Review Board or Ethics Committee Address(es)
1183	Dr. Julio A. Ramirez		Mary Beth Allen<br>Dr. Marty Allen<br>Amirali Ali Amjadi<br>Dr. Forest W. Arnold<br>Uchenna Ezike<br>Rama Kapoor<br>Dr. Pauline A. Lett<br>Dr. Maricar F. Malinis<br>Dr. Raul Nakamatsu<br>Alpa Patel<br>Paula Peyrani<br>Dr. Paul S. Schulz<br>Maria Tillan<br>Inemesit Umoren<br>Cathy Whalen	Ambulatory Care Building<br>550 South Jackson Street<br>Louisville, KY 40202<br>UNITED STATES<br><br>University Hospital<br>530 South Jackson Street<br>Louisville, KY 40202<br>UNITED STATES	University of Louisville<br>Human Subjects Protection Program Office<br>MedCenter One, Suite 200<br>501 East Broadway<br>Louisville, KY 40202<br>UNITED STATES
1184 *	Dr. Cash R. Beechler		Dr. Paul J. Conomos<br>Dr. James E. Mojica<br>Dr. Paul Terry Steinmetz<br>Arthur J. Tillinghast<br>Dr. Karen L. Wright	Scottsdale HealthCare Shea<br>9003 East Shea Blvd.<br>Scottsdale, AZ 85260<br>UNITED STATES	Scottsdale Healthcare Shea<br>Institutional Review Board<br>9003 East Shea Boulevard<br>Scottsdale, AZ 85260<br>UNITED STATES
1190 *	Dr. Albert H. Olivencia-Yurvati		Dr. Barbara A. Atkinson<br>Dr. Mirza W. Baig<br>Arnold A. Fikkert<br>Mr. Christopher Hayes<br>Dr. Mark Joseph Hupert	Plaza Medical Center of Fort Worth<br>900 8th Avenue<br>Fort Worth, TX 76104<br>UNITED STATES<br><br>UNIVERSITY OF NORTH TEXAS HEALTH SCIENCE CENTER AT FORT WORTH<br>855 MONTGOMERY STREET<br>FORT WORTH, TX 76107<br>UNITED STATES	Plaza Medical Center of Fort Worth<br>Institutional Review Board for the Protection of Human Subjects<br>900 Eight Avenue<br>Fort Worth, TX 76104<br>UNITED STATES<br><br>University of North Texas Health Science Center at Fort Worth, Texas College of Osteopathic Medicine<br>Institutional Review Board for the Protection of Human Subjects<br>999 Montgomery Street<br>Fort Worth, TX 76107<br>UNITED STATES

* Did not randomize subjects

090177e1816ca80c\Approved\Approved On: 06-Oct-2010 15:16

Center	Principal Investigator	Co-Investigator(s)	Sub-Investigator(s)	Address(es)	Institutional Review Board or Ethics Committee Address(es)
1197	Dr. Steven Wallace Parker		Donna L. Dominguez<br>Dr. Michael Vincent Jackson<br>Dr. Franklin Kevin Murphy<br>Elaine M. Yee	Pulmonary Medicine Associates<br>Suite 801<br>236 W. 6th Street<br>Reno, NV 89503<br>UNITED STATES<br><br>Renown Regional Medical Center<br>1155 Mill Street<br>Reno, NV 89502<br>UNITED STATES<br><br>Sierra Infectious Diseases<br>Suite 705<br>75 Pringle Way<br>Reno, NV 89502<br>UNITED STATES	Renown Regional Medical Center<br>Institutional Review Board<br>Mail Stop X-19<br>1155 Mill Street<br>Reno, NV 89502<br>UNITED STATES
1199	Dr. Lee Edward Morrow		Kristi L. Farrington<br>Amy A. Keyes<br>Dr. Richard D. Le<br>Lori D. Mahon<br>Mark A. Malesker<br>Francisco A. Romero Jr.<br>Dr. Dan Schuller<br>Dr. Robert E. Wear III<br>Dr. Tammy O. Wichman	Creighton University Medical Center<br>601 North 30th Street<br>Omaha, NE 68131<br>UNITED STATES	Creighton University Institutional Review Board<br>Research Compliance Office<br>2500 California Plaza<br>Omaha, NE 68178<br>UNITED STATES

090177e1816ca80c\Approved\Approved On: 06-Oct-2010 15:16

Center	Principal Investigator	Co-Investigator(s)	Sub-Investigator(s)	Address(es)	Institutional Review Board or Ethics Committee Address(es)
1200	Dr. Gyorgy Frendl		Hina Alam<br>Dr. Rodrigo F. Alban Barba<br>Dr. Edward R. Garcia<br>Syeda Z. Gardezi<br>Sean Garvin<br>Dr. William B. Gormley<br>Dr. James P. Hardy<br>Joaquim Havens<br>Peter C. Hou<br>Dr. Petr Jarolim<br>Edward Kelly<br>Barrett T. Kitch<br>Dr. Anthony F. Massaro<br>Dr. Kimberly Matzie<br>Dr. Shannon Sue McKenna<br>Chad M. Meyers<br>Dr. Fani Nhuch<br>Dr. David Oxman<br>Arvind Palanisamy<br>Dr. James D. Rawn<br>Amy L. Rezak<br>Dr. Selwyn O. Rogers<br>Dr. Nicholas Sadovnikoff<br>Naomi Shimizu<br>James F. Watkins<br>Dr. Gerald L. Weinhouse<br>Dr. Maxwell Weinmann<br>Dr. Mallory Williams<br>Dr. Joshua H. Winer	Brigham and Women's Hospital<br>75 Francis Street<br>Boston, MA 02115<br>UNITED STATES	Partners Human Research Committee<br>Partners Human Research Office<br>Suite 1002<br>116 Huntington Avenue<br>Boston, MA 02116<br>UNITED STATES
1201 *	Dr. Andrew F. Shorr		Dr. Nazli Bolouri<br>Dr. Chee Chan<br>Dr. Daniel L. Herr<br>Pejman Kharazi<br>Mary Margaret Lewis<br>Claude William Nogay<br>martin pinkett	Washington Hospital Center<br>110 Irving Street, NW<br>Washington, DC 20010<br>UNITED STATES	Medstar Research Institute<br>Institutional Review Board<br>Suite 201<br>6495 New Hampshire Avenue<br>Hyattsville, MD 20783<br>UNITED STATES

* Did not randomize subjects

090177e1816ca80c\Approved\Approved On: 06-Oct-2010 15:16

Center	Principal Investigator	Co-Investigator(s)	Sub-Investigator(s)	Address(es)	Institutional Review Board or Ethics Committee Address(es)
1208	Dr. Adel M. Bassily-Marcus		Dr. Ernest Benjamin<br>Rosanna Del Giudice<br>Evan Grolley<br>Dr. Roopa Kohli-Seth<br>Andrew B. Leibowitz<br>Dr. Steven Lin<br>Dr. Anthony Robert Manasia<br>Dr. John Mark Oropello	Mount Sinai Medical Center<br>One Gustave L. Levy Place<br>New York, NY 10029<br>UNITED STATES	Mount Sinai School of Medicine<br>Institutional Review Board<br>Box 1075<br>One Gustave L. Levy Place<br>Icahn Medical Institute, Building 4-78<br>New York, NY 10029<br>UNITED STATES
1218	Dr. Roy C. St. John		Joan C. Cook<br>Erin M. Gedling	Columbus Pulmonary and Critical Care<br>Suite 610<br>745 West State Street<br>Columbus, OH 43222<br>UNITED STATES<br><br>Mount Carmel West Hospital<br>793 West State Street<br>Columbus, OH 43222<br>UNITED STATES<br><br>Remington-Davis, Inc.<br>Suite 106A<br>1335 Dublin Road<br>Columbus, OH 43215<br>UNITED STATES	Mount Carmel Health System<br>Human Subject Protection Office, Corporate Services Center<br>6150 East Broad Street<br>Columbus, OH 43213<br>UNITED STATES

* Did not randomize subjects

090177e1816ca80c\Approved\Approved On: 06-Oct-2010 15:16

Center	Principal Investigator	Co-Investigator(s)	Sub-Investigator(s)	Address(es)	Institutional Review Board or Ethics Committee Address(es)
1219	Dr. Luis Ernesto Jauregui Peredo		Dr. Arlette Tanos Aouad<br>Pamela H. Beavers<br>Charles M. Bork<br>Dr. Tanyanyiwa W. Chinyadza<br>Lisa S. Graham<br>Dr. Srinivas Katragadda<br>Dr. Vijay Kumar Mahajan<br>Mary M. McCormick<br>Ms. Kristen Kay Zaiger Miller<br>Dr. Nelson Nicolasora<br>Darla J. Scott<br>Mary E. Scott<br>Tina M. Steinhauser<br>Dr. Darrell Wayne Stuart<br>Dee Ann Tilley<br>Dr. James Anthony Tita	ID Clinical Research, LTD<br>Suite 203<br>2409 Cherry Street<br>Toledo, OH 43608<br>UNITED STATES<br><br>Infectious Disease Associates of Northwest Ohio, Inc.<br>#1400<br>2222 Cherry Street<br>Toledo, OH 43608<br>UNITED STATES<br><br>Regency Hospital<br>5220 West Alexis Road<br>Sylvania, OH 43560<br>UNITED STATES<br><br>St. Vincent Mercy Medical Center<br>2213 Cherry Street<br>Toledo, OH 43608<br>UNITED STATES	St. Vincent Mercy Medical Center<br>Institutional Review Board<br>2213 Cherry Street<br>Toledo, OH 43608<br>UNITED STATES
1220	Dr. Luca M. Bigatello		Houman Amirfarzan<br>Dr. Robert S. Harris<br>Dr. Judith Hellman<br>Kanya Kumwilaisak<br>Silvia Pivi<br>Dr. Ulrich H. Schmidt	Massachusetts General Hospital<br>Clinics 309<br>55 Fruit Street<br>Boston, MA 02114<br>UNITED STATES	Partners Human Research Committee<br>Partners Human Research Office<br>Suite 1002<br>116 Huntington Avenue<br>Boston, MA 02116<br>UNITED STATES
1229 *	Dr. Marc J. Shapiro		Eileen Finnin<br>Debbie A. Fitzgerald<br>Dr. Michael F. Paccione<br>Wayne Patterson<br>Steven Sandoval	Stony Brook University Hospital<br>101 Nicolls Road<br>Stony Brook, NY 11794<br>UNITED STATES	Stony Brook University Institutional Review Board<br>Office of Vice President of Research<br>Research Information and Compliance<br>Melville Library Fifth Floor<br>Stony Brook, NY 11794<br>UNITED STATES

* Did not randomize subjects

090177e1816ca80c\Approved\Approved On: 06-Oct-2010 15:16

Center	Principal Investigator	Co-Investigator(s)	Sub-Investigator(s)	Address(es)	Institutional Review Board or Ethics Committee Address(es)
1230	Dr. Therese M. Duane		Michel Badih Aboutanos<br>Christopher Todd Borchers<br>Dr. Rao R. Ivatury<br>Lori B. Kei<br>Dr. Ajai Kumar Malhotra<br>Robin Gregory Sculthorpe	Virginia Commonwealth University Medical Center, Div. of Trauma, Critical Care & Emergency Medicine<br>West Hospital, 15th Floor<br>1200 East Broad Street<br>Richmond, VA 23219<br>UNITED STATES	Western Institutional Review Board, Inc.<br>3535 Seventh Avenue SW<br>Olympia, WA 98502-5010<br>UNITED STATES
1231	Dr. Edgar J. Jimenez		Dr. Philip Anthony Giordano<br>Dr. Rakesh C. Gupta<br>Samantha Munro<br>Christina Robinson<br>Dr. Carlos A. Ruiz<br>Dr. Orlando Ismael Ruiz Rodriquez<br>Dr. Jeffrey Adam Sadowsky<br>Jean Schiller<br>Donald C. Vickers<br>Laura H. Waite<br>Dr. Kurt D. Weber<br>Dr. Christian C Zuver	Lucerne Hospital<br>818 Main Lane<br><br>Orlando, FL 32801<br>UNITED STATES<br><br>Orlando Regional Medical Center<br>1414 Kuhl Avenue<br>Orlando, FL 32806<br>UNITED STATES	Orlando Health Institutional Review Board<br>1414 Kuhl Avenue<br><br>Orlando, FL 32806<br>UNITED STATES

090177e1816ca80c\Approved\Approved On: 06-Oct-2010 15:16

Center	Principal Investigator	Co-Investigator(s)	Sub-Investigator(s)	Address(es)	Institutional Review Board or Ethics Committee Address(es)
1237	Elamin Muhamoud Elamin		Dr. Susan E. Beltz<br>Dr. Azra Bihorac<br>Dr. Sarah Elizabeth Bush<br>Dr. Lawrence Joseph Caruso<br>Dr. Marlena Allison Fox<br>Dr. Andrea Gabrielli<br>Dr. Thomas James Gallagher<br>Dr. Deborah Ann Kahler<br>Dr. Abraham Joseph Layon<br>Dr. Aimee Christine LeClaire<br>Dr. Larry Christopher Martin<br>Dr. Mona K. Patel<br>Dr. Carl W. Peters<br>Dr. Margaret Ross Thomson<br>Dr. Angela R. Wills	Shands Hospital University of Florida<br>1600 Southwest Archer Road<br>Gainesville, FL 32610<br>UNITED STATES	Western Institutional Review Board<br>PO Box 12029<br>3535 Seventh Avenue, SW<br>Olympia, WA 98502<br>UNITED STATES
1239	Dr. Miguel M. Mogyoros		Dr. Jennifer Biltoft	Exempla Saint Joseph Hospital<br>1835 Franklin Street<br>Denver, CO 80218<br>UNITED STATES<br><br>Kaiser Permanante<br>20th Avenue Medical Center<br>2045 Franklin Street<br>Denver, CO 80205<br>UNITED STATES	Exempla Healthcare Institutional Review Board<br>1835 Franklin Street<br>Denver, CO 80218<br>UNITED STATES<br><br>Kaiser Permanente Institutional Review Board<br>Suite 300<br>10065 East Harvard Avenue<br>Denver, CO 80231<br>UNITED STATES

090177e1816ca80c\Approved\Approved On: 06-Oct-2010 15:16

Center	Principal Investigator	Co-Investigator(s)	Sub-Investigator(s)	Address(es)	Institutional Review Board or Ethics Committee Address(es)
1244	Dr. Alejandro C. Arroliga		Faith S. Rothermel<br>Dr. Christopher D. Spradley	Scott and White Memorial Hospital<br>2401 South 31st Street<br>Temple, TX 76508<br>UNITED STATES	Scott and White Memorial Hospital<br>Institutional Review Board<br>2401 South 31st Street<br>Temple, TX 76508<br>UNITED STATES
1245	Dr. Charles A. Adams Jr.		Steven Benkert<br>Michael Connolly<br>Dr. Kevin Maier Dushay<br>Shea Gregg<br>David T. Harrington<br>David Heffernan<br>Dr. James Raymond Klinger<br>Matthew S. Kozloff<br>Mitchell M. Levy<br>Kenneth A. Lynch<br>Sarah D. Majercik<br>Maria Andrea Monckeberg	Rhode Island Hospital<br>APC 415<br>593 Eddy Street<br>Providence, RI 02903<br>UNITED STATES	Rhode Island Hospital<br>Office of Research Administration<br>Aldrich Building, Fifth Floor<br>593 Eddy Street<br>Providence, RI 02903<br>UNITED STATES

090177e1816ca80c\Approved\Approved On: 06-Oct-2010 15:16

Center	Principal Investigator	Co-Investigator(s)	Sub-Investigator(s)	Address(es)	Institutional Review Board or Ethics Committee Address(es)
1246	Dr. Joseph Barney		Dr. Jennifer Davis<br>Dr. Mark Dransfield<br>Melissa Garner<br>Rebecca Quinn<br>Nancy Stansfield<br>Keith M. Wille	UAB Hospital<br>619 South 19th Street<br>Birmingham, AL 35294<br>UNITED STATES<br><br>UAB Hospital / Russell Pharmacy<br>RAC 117<br>1813 6th Ave<br>Birmingham, AL 35294<br>UNITED STATES<br><br>UAB Lung Health Center<br>526 20th Street South<br>Birmingham, AL 35249<br>UNITED STATES<br><br>University of Alabama at Birmingham Kirklin Clinic<br>2000 Sixth Avenue South<br>Birmingham, AL 35233<br>UNITED STATES	WIRB<br>3535 Seventh Ave. SW<br>Olympia, WA 98502-5010<br>UNITED STATES

090177e1816ca80c\Approved\Approved On: 06-Oct-2010 15:16

Center	Principal Investigator	Co-Investigator(s)	Sub-Investigator(s)	Address(es)	Institutional Review Board or Ethics Committee Address(es)
1249	Dr. Timothy Eugene Albertson		Dr. Roblee Peter Allen<br>Dr. Mark Avdalovic<br>Dr. Andrew Leong Chan<br>Cinda Christensen<br>Jordan Fein<br>Jason Ferries<br>Dr. Kimberly Ann Hardin<br>Dr. Richart Harper<br>Heba A. Ismail<br>Dr. Nicholas James Kenyon<br>Dr. Samuel Louie<br>Dr. Brian Morrissey<br>Anandray Patel<br>Dr. Christian Erik Sandrock<br>Matthew P. Sisitki<br>Dr. Ken Yoneda	UC Davis Medical Center<br>2315 Stockton Blvd<br>Sacramento, CA 95817<br>UNITED STATES	IRB Administration<br>Suite 1400<br>2921 Stockton Boulevard<br>CTSC Building<br>Sacramento, CA 95817<br>UNITED STATES
1250	Dr. Eugene Carl Fletcher Jr.		Krystal Angevine<br>LeAnn Doddridge<br>Joyce Fletcher<br>James Isbell<br>Ryan Pickhardt<br>Judy Sepulveda	Floyd Memorial Hospital<br>1850 State Street<br>New Albany, IN 47151<br>UNITED STATES	Floyd Memorial Hospital and Health Services<br>1850 State Street<br>New Albany, IN 47150<br>UNITED STATES
1251 *	Dr. Rudolf J. Kotula		Dr. Kyle Mills	Swedish Medical Center<br>501 East Hampden Avenue<br>Englewood, CO 80110<br>UNITED STATES	HCA HealthONE Institutional Review Board<br>Suite 265A<br>720 South Colorado Boulevard<br>Glendale, CO 80246<br>UNITED STATES

* Did not randomize subjects

090177e1816ca80c\Approved\Approved On: 06-Oct-2010 15:16

Center	Principal Investigator	Co-Investigator(s)	Sub-Investigator(s)	Address(es)	Institutional Review Board or Ethics Committee Address(es)
1252	Dr. Paul James Carson		Abdulhamid Alkhalaf<br>Augusto Alonto<br>steven briggs<br>Hasrat Khan<br>Regis Lager<br>David McNamara<br>Dr. Robert Nelson<br>Frank Sepe<br>Almothana Shanaah<br>Gino Tapia-Zegarra<br>Mark Tieszen	MeritCare Broadway Health Center<br>736 Broadway North<br>Fargo, ND 58122<br>UNITED STATES<br><br>MeritCare Clinic<br>737 Broadway North<br>Fargo, ND 58122<br>UNITED STATES<br><br>MeritCare Medical Center<br>801 Broadway North<br>Fargo, ND 58112<br>UNITED STATES	MeritCare Health System IRB<br>MeritCare Health System IRB<br>Route 701<br>801 Broadway N<br>Fargo, ND 58122-0701<br>UNITED STATES
1253	Dr. Richard V. Spera		Dr. Edward Eiland<br>Celeste Fletcher<br>Sheryl Guffin<br>Dr. Ali A. M. Hassoun<br>Jason Smith	Alabama Infectious Disease Center, P.C.<br>301<br>420 Lowell Drive, SE<br>Huntsville, AL 35801<br>UNITED STATES<br><br>Huntsville Hospital<br>101 Sivley Road<br>Huntsville, AL 35801<br>UNITED STATES	Huntsville Hospital Institutional Review Committe<br>101 Sivley Road<br>Huntsville, AL 35801<br>UNITED STATES

090177e1816ca80c\Approved\Approved On: 06-Oct-2010 15:16

Center	Principal Investigator	Co-Investigator(s)	Sub-Investigator(s)	Address(es)	Institutional Review Board or Ethics Committee Address(es)
1254	Muhammad Shibli		Jose Bordon<br>oyinlola Fashina	Providence Hospital<br>Suite # 203<br>1160 Varnum Street, NE<br>Washington, DC 20017<br>UNITED STATES<br><br>Providence Hospital<br>Clinical Research Center<br>Room 108 H<br>1150 Varnum Street North East<br>ACC Building<br>Washington, DC 20017<br>UNITED STATES	Western Institutional Review Board, Inc.<br>WIRB<br>3535 Seventh Avenue, Southwest<br>Olympia, WA 98502-5010<br>UNITED STATES
1258	Dr. Richard W. Light		Allie M Bell<br>Anupama G. Brixey<br>Hope Campbell<br>Dr. Alison Miller<br>Hollis O'Neal<br>Dr. Elisabeth Willers	Vanderbilt University<br>Institutional Review Board<br>1313 21st Ave South<br>Nashville, TN 37232<br>UNITED STATES<br><br>Vanderbilt University Medical Center<br>1161 21st Ave<br>Nashville, TN 37232<br>UNITED STATES	Vanderbilt University Institutional Review Board<br>504 Oxford House<br>1313 21st Avenue South<br>Nashville, TN 37232<br>UNITED STATES
1261	Dr. David Conrad Willms		Melissa K. Brown<br>Mihaela Buda<br>Matthew Geriak<br>Dr. Charles Frederick Landers<br>Thomas Lawrie<br>John LeMoine<br>Coletta O'Donnell<br>Richard Sacks<br>Enga M. Santman<br>Dr. Davies Yat-wing Wong	Chest Medicine and Critical Care Medical Group, INC<br>7901 Frost Street<br>San Diego, CA 92123<br>UNITED STATES	Sharp HealthCare IRB Office<br>8695 Spectrum Center Blvd.<br>San Diego, CA 92123<br>UNITED STATES

090177e1816ca80c\Approved\Approved On: 06-Oct-2010 15:16

Center	Principal Investigator	Co-Investigator(s)	Sub-Investigator(s)	Address(es)	Institutional Review Board or Ethics Committee Address(es)
1262 *	Dr. Paul James Manos		Dr. Ken Ray Iwaoka<br>Kia Lee<br>Alison J. McManus<br>Dr. William Dabney ORiordan<br>Andrew Nathan Ramirez	eStudySite-Tri City Medical Center<br>Suite F<br>3998 Vista Way<br>Oceanside, CA 92056<br>UNITED STATES	Western Institutional Review Board, Inc.<br>3535 Seventh Avenue, SouthWest<br>Olympia, WA 98502<br>UNITED STATES
1263 *	Dr. Jan Alan Winetz		Lizbeth Flores-Byrne<br>Ms. Emily Foo<br>Dr. William Dabney ORiordan	eStudySite<br>Good Samaritan Hospital<br>Suite 504<br>2505 Samaritan Drive<br>San Jose, CA 95124<br>UNITED STATES<br><br>Good Samaritan Hospital<br>2425 Samaritan Drive<br>San Jose, CA 95124<br>UNITED STATES<br><br>Good Samaritan Research Office<br>Suite 504<br>2505 Samaritan Drive<br>San Jose, CA 95124<br>UNITED STATES	WESTERN IRB<br>WESTERN IRB<br>3535 7TH AVENUE SW<br>OLYMPIA, WA 98502<br>UNITED STATES

090177e1816ca80c\Approved\Approved On: 06-Oct-2010 15:16

* Did not randomize subjects

Center	Principal Investigator	Co-Investigator(s)	Sub-Investigator(s)	Address(es)	Institutional Review Board or Ethics Committee Address(es)
1264	Dr. Antonio Ramirez Anzueto		Dr. Sandra G. Adams<br>Marc Chalaby<br>Virginia Doyal<br>Michael John Gass<br>Jennifer Hillman<br>Timothy Houlihan<br>Stephanie Levine<br>Carlos Orozco<br>Jay Peters<br>Dr. Marcos Ignacio Restrepo<br>Juan F. Sanchez	South Texas Veterans Health Care System<br>AUDIE L MURPHY DIVISION<br>Pulmonary Disases<br>Mail Code 111E<br>7400 Merton-Minter Boulevard<br>San Antonio, TX 78284<br>UNITED STATES<br><br>University Hospital<br>4502 Medical Drive<br>San Antonio, TX 78229<br>UNITED STATES<br><br>University of Texas Health Care Sciences San Antonio<br>7703 Floyd Curl Drive<br>San Antonio, TX 78229<br>UNITED STATES	University of Texas Health Care Sciences San Antonio<br>Institutional Review Board (MC 7830)<br>7703 Floyd Curl Drive<br>San Antonio, TX 78229<br>UNITED STATES
1265	Dr. Gerard James Fulda		Jaime Cesar Giraldo Arango<br>Mark D. Cipolle<br>Lauren Danese<br>Dr. Michael R. Depietro<br>Dr. Frederick Alan Giberson<br>Steven A. Johnson<br>Michael Kalina<br>Dr. Michael Rhodes<br>Dr. Glenn Herman Tinkoff	Christiana Care Health Services<br>4755 Ogletown Stanton Road<br>Newark, DE 19718<br>UNITED STATES	Christiana Care Health System<br>Institutional Review Board<br>Room 3400<br>501 W. 14th Street<br>Wilmington, DE 19899<br>UNITED STATES

090177e1816ca80c\Approved\Approved On: 06-Oct-2010 15:16

090177e1816ca80c\Approved\Approved On: 06-Oct-2010 15:16

Center	Principal Investigator	Co-Investigator(s)	Sub-Investigator(s)	Address(es)	Institutional Review Board or Ethics Committee Address(es)
1266 *	Dr. Lyssette de L. Cardona Bonet		Javier Gonzalez<br>Dr. Jose Ramirez<br>Leira Ramira Ruiz<br>Griorgio Tarchini	Cleveland Clinic Florida<br>2950 Cleveland Clinic Blvd<br>Weston, FL 33331<br>UNITED STATES<br><br>Cleveland Clinic Florida Hospital<br>3100 Weston Road<br>Weston, Fl 33331<br>UNITED STATES	Cleveland Clinic Florida Institutional Review Board<br>2950 Cleveland Clinic Boulevard<br>Weston, FL 33331<br>UNITED STATES
1267	Dr. Ali Albert El-Solh		Dr. Karin A. Provost<br>Dr. Sanjay Sethi	Veterans Affairs of Western New York Health Care System<br>3495 Bailey Ave<br>Buffalo, NY 14215<br>UNITED STATES	Veterans Affairs Of Western New York Healthcare System<br>3495 Bailey Avenue<br>Buffalo, NY 14215<br>UNITED STATES
1268	Dr. Firas A. Koura		Michael W. Raichel	Hazard ARH Regional Medical Center<br><br>100 Medical Center Drive<br>Hazard, KY 41701<br>UNITED STATES	Appalachian Regional Heather Care Inc IRB<br>100 Airport Gardens Road<br>Hazard, KY 41701<br>UNITED STATES
1270	Dr. Michael R. Silver		Dr. Robert Allen Balk<br>Joyce D. Brown<br>Dr. Larry C. Casey<br>Christopher Crank<br>Dr. David P. Gurka<br>Dr. Omar Lateef<br>Gail Ruderman<br>Dr. Rajive Tandon<br>Dr. Mark Yoder	Rush University Medical Center<br>1653 West Congress Parkway<br>Chicago, IL 60612<br>UNITED STATES<br><br>Rush University Medical Center<br>Pulmonary Outpatient Clinic<br>Suite 050<br>1725 West Harrison<br>Chicago, IL 60612<br>UNITED STATES	Rush University Medical Center Research and Clinical Trials Main Office<br>1653 West Congress Parkway<br>Chicago, IL 60612<br>UNITED STATES

* Did not randomize subjects

Center	Principal Investigator	Co-Investigator(s)	Sub-Investigator(s)	Address(es)	Institutional Review Board or Ethics Committee Address(es)
1271	Dr. Nathan C. Dean		Dr. Ali Ahmed<br>Anita Austin<br>Dr. Samuel Brown<br>Dr. David Dienhart<br>Heather Gallo<br>Dr. Edgar Garcia-Morales<br>Dr. Mark Goddard<br>Dr. Colin Grissom<br>Dr. Eliotte Hirshberg<br>Naresh Kumar<br>Dr. Russell Miller III.<br>Robert Muelleck<br>Dr. Patricia Nelson<br>Dr. James Orme Jr.<br>Dr. Sunny Pandita<br>Dr. Dean Roller<br>Leanne Struck<br>Dr. Don Van Boerum<br>Dr. Thomas White<br>Dr. Mark Zenger<br>Dr. John Zurasky	Intermountain Healthcare<br>Department of Pulmonary and Critical Care Medicine<br>5121 S. Cottonwood Drive<br>Murray, UT 84157<br>UNITED STATES	Intermountain Healthcare<br>Intermountain Healthcare Urban Central Region IRB<br>LDS Hospital<br>8th Avenue and C Street<br>Salt Lake City, UT 84143<br>UNITED STATES
1272	Adam Matthew Bressler		vorlak Hong<br>Dr. Avery Howard Nathanson<br>Mark T. Pollock<br>Woratute Supaongprapa	Atlanta Institute for Medical Research, Incorporated<br>Suite 220<br>495 Winn Way<br>Decatur, GA 30030<br>UNITED STATES<br><br>Dekalb Medical<br>2701 North Decatur Road<br>Decatur, GA 30033<br>UNITED STATES	DeKalb Medical Center Institutional Review Board<br>2701 North Decatur Road<br>Decatur, GA 30033<br>UNITED STATES
1274	Nicholas J. Pastis		Tanna Cooper<br>Joe Mazur<br>Dr. Charlton Strange	Medical University of South Carolina<br>96 Jonathan Lucas Street<br>Charleston, SC 29425<br>UNITED STATES	Medical University IRB<br>Suite 601, MSC 857<br>19 Hagood Ave<br>Charleston, SC 29425<br>UNITED STATES

* Did not randomize subjects

090177e1816ca80c\Approved\Approved On: 06-Oct-2010 15:16

Center	Principal Investigator	Co-Investigator(s)	Sub-Investigator(s)	Address(es)	Institutional Review Board or Ethics Committee Address(es)
1275	Michael H. Metzler		Dr. Ronald A. Shockley<br>Sheri Stucke	Sunrise Hospital And Medical Center<br>3186 South Maryland Parkway<br>Las Vegas, NV 89109<br>UNITED STATES	Sunrise Instibutional Reveiw Board<br>3186 South Maryland Parkway<br>Las Vegas, NV 81909<br>UNITED STATES
1276	Dr. Philip M. Alapat		Dr. Bindu Akkanti<br>Dr. Venkata D. Bandi<br>Dr. Goutham Dronavalli<br>Dr. Kalpalatha Kummamuri Guntupalli<br>Dr. Elizabeth S. Guy<br>Dr. Nicola Alexander Hanania<br>Dr. Antara Mallampalli	Ben Taub General Hospital<br>1504 Taub Loop<br>Houston, TX 77030-1608<br>UNITED STATES	Institutional Review Board for Human Subject Research<br>Baylor College of Medicine and Affiliated Hospitals<br>BCM 310<br>One Baylor Plaza<br>Houston, TX 77030<br>UNITED STATES
1278	Dr. Glen Bouder		Carol Hemmen<br>Benjamin Franklin Lewis<br>Jeffrey Spray	Winchester Medical Center<br>1840 Amherst Street<br>Winchester, VA 22601<br>UNITED STATES	Winchester Medical Center<br>Institutional Review Board<br>1840 Amherst Street<br>Winchester, VA 22601<br>UNITED STATES
1279 *	Dr. William Patrick Tillis		Dr. Rosario Ruth Avelino<br>Rajesh Balagani<br>Subramanyam Chittivelu<br>Dr. Jeremiah Edward Crabb VI<br>Athir Hajjar<br>Ms. Kimberly L. Hartwig<br>Dr. Michael Leroy Peil<br>Dr. William Anthony Sauder<br>Ashley L. Scott<br>Harbhajan Singh<br>Dr. Patrick Eugene Whitten	Illinois Lung and Critical Care Institute<br>Suite 200<br>1001 Main Street<br>Peroia, IL 61606<br>UNITED STATES<br><br>Saint Francis Medical Center<br>530 NorthEast Glen Oak Avenue<br>Peroia, IL 61637<br>UNITED STATES	University of Illinois College of Medicine at Peoria Institutional Review Board<br>Box 1649<br>One Illini Drive<br>Peoria, IL 61656-1649<br>UNITED STATES

* Did not randomize subjects

090177e1816ca80c\Approved\Approved On: 06-Oct-2010 15:16

Center	Principal Investigator	Co-Investigator(s)	Sub-Investigator(s)	Address(es)	Institutional Review Board or Ethics Committee Address(es)
1285	Dr. Ernest Eugene Moore Jr.		Lee Anne Ammons<br>Carlton Carter Barnet<br>Walter L. Biffl<br>Catherine Cothren<br>Laura Fahrenbrook<br>James B. Haenel<br>Jeffrey Leon Johnson<br>Gervaise Edward Kimm Jr.	Denver Health Medical Center<br>Department of Surgery<br>777 Bannock Street<br>Denver, CO 80204<br>UNITED STATES	University of Colorado<br>13001 E. 17th Place<br>Building 500, Rm N3214<br>Aurora, CO 80010<br>UNITED STATES<br><br>Western Institutional Review Board, Inc.<br>3535 Seventh Ave SW<br>Olympia, WA 98508<br>UNITED STATES

090177e1816ca80c\Approved\Approved On: 06-Oct-2010 15:16

Venezuela

Coordinating Investigators:
Dr. Dolores Moreno
Dra. Bernardina Sumoza

Center	Principal Investigator	Co-Investigator(s)	Sub-Investigator(s)	Address(es)	Institutional Review Board or Ethics Committee Address(es)
1053 *	Dr. Gur Yehuda Levy		Carlos Talamo	Hospital Universitario de Caracas<br>Servicio de Neumonología. Piso 8.<br>Los Chaguaramos<br>Universidad Central de Venezuela. UCV.<br>Caracas, Distrito Capital 1041<br>VENEZUELA	Instituto Autónomo. Hospital Universitario de Caracas<br>Comité de Ética.<br>Universidad Central de Venezuela. UCV<br>Los Chaguaramos<br>Caracas, Distriti Capital 1041<br>VENEZUELA
1055 *	Dr. Jairo Pacheco		Dr. Orlando Nava	Hospital Clínico de Maracaibo<br>Servicio de Infectología<br>Avenida 15 con Calle 59<br>Urbanización Trinidad.<br>Prolongación Delicias<br>Maracaibo, Estado Zulia 4002<br>VENEZUELA	Comité de Ética del Hospital Clínico de Maracaibo.<br>Comité de Ética Hospital Clínico de Maracaibo<br>Avenida 15 con Calle 59.<br>Urbanización La Trinidad.<br>Prolongación Delicias.<br>Maracaibo, Estado Zulia 4002<br>VENEZUELA

* Did not randomize subjects

090177e1816ca80c\Approved\Approved On: 06-Oct-2010 15:16
